# Elasticity-based boosting of neuroepithelial nucleokinesis via indirect energy transfer from mother to daughter

**DOI:** 10.1371/journal.pbio.2004426

**Published:** 2018-04-20

**Authors:** Tomoyasu Shinoda, Arata Nagasaka, Yasuhiro Inoue, Ryo Higuchi, Yoshiaki Minami, Kagayaki Kato, Makoto Suzuki, Takefumi Kondo, Takumi Kawaue, Kanako Saito, Naoto Ueno, Yugo Fukazawa, Masaharu Nagayama, Takashi Miura, Taiji Adachi, Takaki Miyata

**Affiliations:** 1 Department of Anatomy and Cell Biology, Nagoya University Graduate School of Medicine, Nagoya, Japan; 2 Department of Biosystems Science, Institute for Frontier Life and Medical Sciences, Kyoto University, Kyoto, Japan; 3 Research Institute for Electronic Science, Hokkaido University, Hokkaido, Japan; 4 Department of Imaging Science, Center for Novel Science Initiatives, National institute for Basic Biology, Okazaki, Japan; 5 Division of Morphogenesis, National institute for Basic Biology, Okazaki, Japan; 6 Laboratory for Morphogenetic Signaling, RIKEN Center for Developmental Biology, Kobe, Japan; 7 Division of Cell Biology and Neuroscience, Faculty of Medical Sciences, University of Fukui, Fukui, Japan; 8 Department of Anatomy and Cell Biology, Graduate School of Medical Sciences, Kyushu University, Fukuoka, Japan; University of Lyon, France

## Abstract

Neural progenitor cells (NPCs), which are apicobasally elongated and densely packed in the developing brain, systematically move their nuclei/somata in a cell cycle–dependent manner, called interkinetic nuclear migration (IKNM): apical during G2 and basal during G1. Although intracellular molecular mechanisms of individual IKNM have been explored, how heterogeneous IKNMs are collectively coordinated is unknown. Our quantitative cell-biological and in silico analyses revealed that tissue elasticity mechanically assists an initial step of basalward IKNM. When the soma of an M-phase progenitor cell rounds up using actomyosin within the subapical space, a microzone within 10 μm from the surface, which is compressed and elastic because of the apical surface’s contractility, laterally pushes the densely neighboring processes of non–M-phase cells. The pressed processes then recoil centripetally and basally to propel the nuclei/somata of the progenitor’s daughter cells. Thus, indirect neighbor-assisted transfer of mechanical energy from mother to daughter helps efficient brain development.

## Author summary

The development of large brain structures, such as the mammalian cerebral cortex, depends on the continuous and efficient production of cells by neural progenitor cells. Neural progenitor cells are elongated and span the developing brain wall. The nuclei and bodies of these cells move cyclically between the apical and basal surfaces, and they divide every time they reach the apical surface. While we understand how individual cells achieve this cycle, how the movements of several progenitor cells are coordinated with one another remains elusive. By using a combination of live imaging and mechanical experiments, coupled with mathematical simulations, we show that cell crowding at the apical surface, where progenitor cells divide, creates a subapical microzone that is compressed and elastic. We then show that when each mother cell rounds up, preparing for division, it pushes this elastic microzone laterally, thereby storing mechanical energy. After cell division, this mechanical energy is transferred to the daughter cells, propelling them along the axis of movement in the direction of the basal surface, in an energy-saving manner. Our mathematical simulations show that timely departure of newly generated daughter cells is critical for the overall tissue structure of the cerebral proliferative zone.

## Introduction

**Fig 1:**
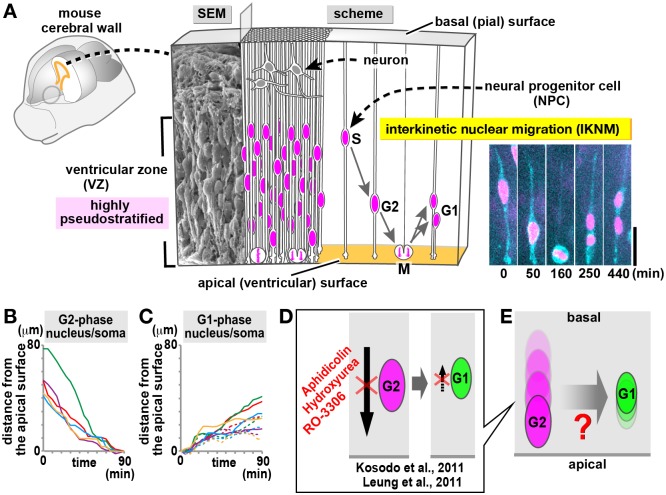
What is the unknown cellular and mechanical mechanism that enables the passive IKNM in the proliferative zone of the embryonic cerebral wall? (A) Schematic representation of the embryonic mouse cerebral wall subjected to the present study. SEM picture of a cerebral wall isolated from an E13 mouse is combined with a cross-sectional scheme (right) depicting NPCs spanning apicobasally across the wall and neurons accumulating in the outer zone. While the outer territory composed of differentiated neurons is really stratified, the inner progenitor territory, called the ventricular zone, is “pseudostratified,” with each nucleus migrating in a cell cycle–dependent manner within an elongated progenitor cell. As exemplified in a clone sparsely labeled (green, plasma membrane; magenta, nucleus) in slice culture, nuclear/somal movement is apical during G2 phase (progenitor, until 160 min) and basal during G1 phase (daughter cells, until 440 min), whereas mitosis occurs at the apical surface (160 min) (see also S1 Movie). These movements are collectively referred to as interkinetic nuclear migration (IKNM). Scale, 20 μm. (B and C) Trajectories of nuclei/somata that migrated apically (B) or basally (C). Modified from Okamoto et al. [18], which is consistent with other previous observations [18–21, 23–26]. (D) Previous studies by Leung et al. [25] and Kosodo et al. [26] showed that pharmacological inhibition of the apical nucleokinesis during G2 resulted in retarded basal nucleokinesis during G1. (E) Current understanding that apicalward nuclear flows in the VZ may induce passive basalward nucleokinesis of G1-phase cells. By which cellular mechanisms this hypothetical passive nucleokinesis occurs is unknown. E, embryonic day; IKNM, interkinetic nuclear migration; NPC, neural progenitor cell; SEM, scanning electron microscopy; VZ, ventricular zone.

Formation of large brains such as the mammalian cerebral cortex depends on continuous and efficient cell production by neural progenitor cells (NPCs) [1, 2, 3]. NPCs are elongated, spanning a developing brain wall (up to 300 μm thick in mice), and divide at the apical surface [4, 5, 6, 7, 8] (Fig 1A). They individually move the nuclei and somata in a cell cycle–dependent manner (apical during G2 and basal during G1), and these nuclear/somal movements are called interkinetic nuclear migration (IKNM) [9, 10, 11, 12] (Fig 1A–1C, S1 Movie). Because IKNMs of different NPCs are not synchronized, IKNM-undergoing regions (100 μm thick, called the neuroepithelium [NE] or ventricular zone [VZ]) display nuclei/somata at diverse apicobasal positions, a histological appearance referred to as pseudostratification (Fig 1A). The degree of neuroepithelial pseudostratification (i.e., the thickness of NE/VZ) increases from mice to monkeys [13] to humans [14, 15]. Extensive pseudostratification is thought to contribute to increasing the frequency of cell divisions per unit apical surface [16]. However, the accompanying dense countercurrents (i.e., apical and basal IKNMs) run the risk of “traffic jams” [17]. For example, experimentally induced failures in basal nucleokinesis lead to near-apical nuclear/somal overcrowding and abnormal brain histogenesis [18]. Despite recent advances in understanding intracellular molecular mechanisms that move the nucleus apically or basally in each NPC [19, 20, 21, 22, 23], how heterogeneous IKNMs are safely and efficiently organized (or horizontally bundled) into an ordered VZ remains unknown. In the embryonic cerebral cortex, cell production (i.e., M-phase NPCs’ divisions) accompanied by massive flows of the “material” (somata of G2-phase NPCs) and the “product” (those of G1-phase NPCs) occurs most extensively and continuously among a wide variety of developing tissues. The high pseudostratification of the cerebral cortical VZs has led to it becoming a model system to study “production logistics” in organogenesis.

To understand a system-level device to make the intra-VZ nuclear/somal traffic as efficient and energy saving as possible, we sought to determine how active and passive movements are coordinated (or collaborate with each other). Previous quantitative imaging studies revealed that apical nucleokinesis during G2 phase (Fig 1B) was faster than basal nucleokinesis during G1 phase (Fig 1C) [18–21, 23–26] and judged to be more directional, with a superlinear mean-squared displacement (MSD) pattern, than more fluctuating basal nucleokinesis showing a linear MSD pattern [18, 21, 25, 27]). When apical nucleokinesis was inhibited through blocking NPCs’ entrance into G2 phase, basal nucleokinesis was retarded (Fig 1D) [25, 26]. These results therefore suggested that apicalward nuclear flows in the VZ may induce passive basalward nucleokinesis of G1-phase cells (Fig 1E). However, the cell–cell interactions that would mechanically contribute to such a hypothetical passive mechanism remained unknown. Especially, how G2-phase cells’ nuclear/somal movement towards/to the apical surface can induce or lead to such hypothetical passive basal nucleokinesis of early G1-phase cells has not been studied. We here show that mechanical energy provided by an apically dividing (M-phase) NPC is transiently stored in the surrounding subapical space (within 10 μm from the surface) and elastically returned to its daughter cells, promoting passive basalward nucleokinesis. The elasticity in the subapical space is established by physiological cell crowding, in which neighboring M-phase and non–M-phase cells collaborate mechanically, thereby enabling energy-efficient IKNMs and benefiting the systematic “production logistics” in the VZ.

## Results

### Cellular composition, dynamics, and elastic properties of the subapical space in mammalian neuroepithelia suggest a new “non-collision” model of passive nuclear migration

**Fig 2:**
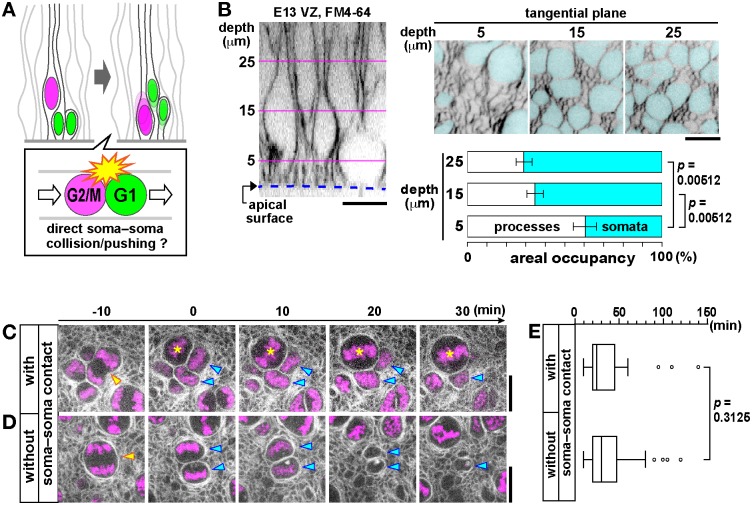
Direct soma–soma collision model not supported in the subapical space. (A) A model for passive launching of newborn G1-phase cells’ nuclei/somata away from the apical surface by direct soma–soma collision/pushing in the subapical space. (B) Apical-to-basal scanning of FM4-64–labeled cell boundaries in the mid-embryonic mouse cerebral cortical VZ, with nuclei/somata highlighted (cyan), with quantification of the areal occupancy by processes or somata at 5 μm, 15 μm, or 25 μm from the apical surface. (C–E) The time between the mother cell’s division (yellow arrowhead) and departure of daughter cell nuclei (cyan arrowhead) was comparable between the presence (*n* = 36, as in C) and absence (*n* = 54, as in D) of new G2/M-phase cells’ somata in contact/collision (asterisk) (*p* = 0.3125, Mann–Whitney *U* test). Scale, 10 μm in B, C, and D. Underlying data can be found in S1 Data. E, embryonic day; VZ, ventricular zone.

We aimed at elucidating where and how the hypothetical passive nuclear/somal movements are induced in the mouse neocortical VZ (Fig 1E). One possibility is that nuclei/somata of early G1-phase cells (i.e., newly generated daughter cells) in the subapical space (within 10 μm from the apical surface) of the VZ are propelled through direct soma–soma collision or pushing by G2- or M-phase cells (Fig 2A). To evaluate this “direct collision” model that we hypothesized in the subapical space, we first examined the density of somata by visualizing the plasma membranes of all cells (Fig 2B). We found that the subapical space had no extracellular gaps and that apical processes (which appeared as fine meshes in horizontal sections) were more abundant (about 60%) than somata (about 40%), whereas the process/soma ratio decreased in deeper (more basal) VZ regions. Time-lapse observations in horizontal sections of the subapical space (5 μm from the apical surface) (Fig 2C and 2D, S2 and S3 Movies) revealed that direct contacts between somata of newly generated daughter cells and those of neighboring G2/M-phase cells, including slight soma–soma contacts, were observed in only 45% of all divisions (*n* = 80). Importantly, the time that elapsed from the generation of daughter cells to their nuclear departure from the subapical space was similar between cases in which the nuclei/somata of daughter cells had direct contacts with G2- or M-phase somata (Fig 2C) and those without such soma–soma contacts (i.e., cases in which the nuclei/somata of daughter cells were neighbored by apical processes, Fig 2D) (Fig 2E). These results do not support the direct soma–soma collision model in the subapical space.

**Fig 3:**
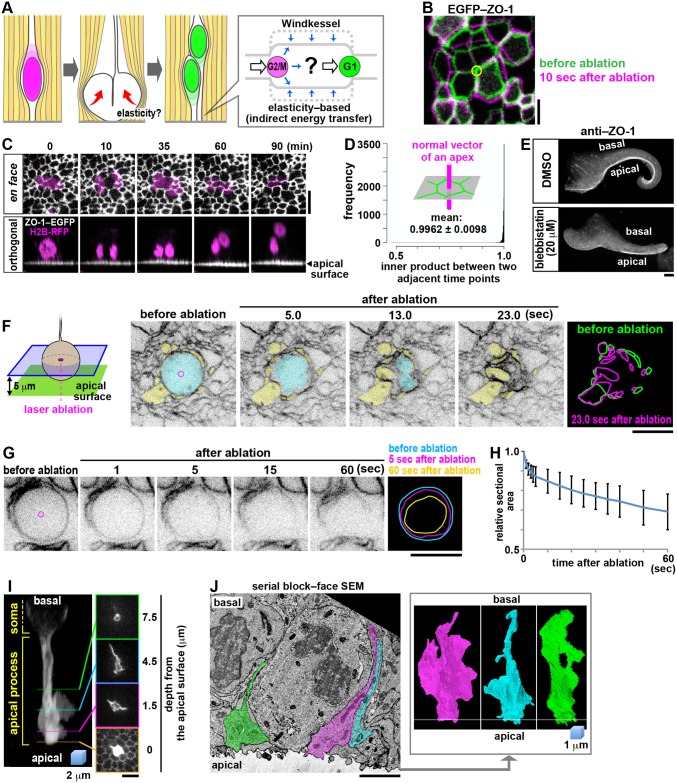
Morphological and mechanical examinations suggest a Windkessel-like elastic property of the subapical space and raise a possibility of elasticity-based nucleokinesis via a mother-to-daughter energy transfer. (A) A model of elasticity-based passive movement of daughter cells’ nuclei/somata (see also S1B and S1C Fig, S4 Movie). (B) En face observation of centrifugal recoiling of laser-ablated apical surface (ablation occurred at the vertex indicated by a yellow circle) EGFP-ZO1–labeled, see also S5 Movie), showing the tangential pretension/contractility of the surface. (C) Time-lapse imaging showing that the apical surface (ZO1-EGFP, white) was stable even at sites of cell division (a clone sporadically labeled with H2B-RFP, magenta) (upper panels, en face projected view; lower panels, orthogonally projected view; see also S6 Movie). (D) Graph showing that “tilting” of apices was minimal (the inner products for the normal vectors to the apices, *x* axis, was close to 1.0) (see also S2A Fig and S7 Movie). (E) Concave-to-convex conversion of apical surface geometry by myosin II inhibition. (F) Centripetal recoiling of the subapical space immediately after laser ablation of an M-phase cell (cyan, the center of the soma was targeted) in a cerebral wall stained with FM4-64 (displacements of the surrounding apical processes [yellow] are summarized in the right panel; see also S8 Movie). (G, H) Quantitative assessment of the change in horizontal sectional area of laser-ablated somata. M-phase cells in cerebral walls sporadically LynN-mCherry–labeled (via in utero electroporation) were ablated at the center of their somata. Graph (H) shows that shrinkage of M-phase somata, as exemplified in F, were consistently reproduced (see also S9 Movie). (I) Three-dimensionally reconstructed live image of an apical process of a slice-cultured VZ cell that dynamically extended lamellipodia-like protrusions only in the subapical space (see also S10 Movie). (J) Serial block-face SEM analysis of the in vivo subapical surface, showing three apical processes (highlighted in a section [left], 3D reconstructed [right]) with lamellipodia-like structures filling the apicalmost (about 5 μm) zone (see also S2J Fig and S12 Movie). Scale, 2 μm in B and J; 5 μm in I; 10 μm in C, F, and G; and 100 μm in E. Underlying data can be found in S1 Data. SEM, scanning electron microscopy; VZ, ventricular zone.

Therefore, we sought to explore an alternative possibility. Even without direct soma–soma collisions, daughter cells’ nuclei/somata could be propelled passively if the surrounding subapical space works like a pressurized air chamber, in which G2-phase cells’ somal influxes and M-phase cells’ voluminous divisions act as an indirect contributor to the displacement of daughter cells’ nuclei/somata (Fig 3A). By analogy, the aorta acts like a compressed air chamber (“Windkessel”) to elastically recoil and forward blood ejected by the heart [28] (S1A Fig). If the subapical space were similarly elastic, as predicted by recent simulation studies [18, 29], mechanical force provided centrifugally by a given mother M-phase cell while expanding to round up would result in storage of elastic energy in the surrounding process-rich subapical space. The stored elastic energy could then be released and returned centripetally to the nuclei/somata of daughter cells generated by that mother cell, thereby externally inducing the daughter cells’ passive nuclear/somal displacement in the basal direction (Fig 3A, S1B and S1C Fig, S4 Movie), just as the aorta forwards blood in a Windkessel-like manner.

Laser ablation on the apical surface resulted in centrifugal recoiling of the surface from the ablated vertex, indicating that it was tangentially contractile (under tension) (Fig 3B and S5 Movie). Quantitative monitoring further revealed that the apical meshwork was quite stable even at sites of cell division, with no local tilting or floppiness (Fig 3C and 3D, S2A Fig, S6 and S7 Movies). Inhibition of actomyosin to diminish the contractility of the apical surface changed the original apically concave morphology of cerebral walls to an apically convex shape (Fig 3E). This pharmacologically induced recoiling response (i.e., the expansion of the subapical space relative to its original volume) suggests that the subapical space may have originally been laterally compressed, as previously suggested in the chicken midbrain tube [30]. To further test the possibility of lateral compression in the subapical space, we performed a novel laser ablation test targeting the center of the soma (Fig 3F). We reasoned that if a cell’s soma under centripetal compression by the surrounding space is ablated to reduce its original physical stability, it would lead to shrinkage of the ablated soma and centripetal displacements of elements in the surrounding space. Subapical laser ablation of M-phase cells’ somata in normal/untreated (apically concave) cerebral walls resulted in a quick centripetal shift of the surrounding processes (Fig 3F and 3G, S8 and S9 Movies). This soma-directed laser ablation quantitatively showed that the subapical space was under compression and ready to recoil (Fig 3G and 3H).

While fiber-like apical processes are flexible and can be bent [18], they are individually under tension along the apicobasal axis [31]. We observed that apical processes contain microtubules bundles, which provide the structural basis for flexural rigidity [32–35], most densely in the region near the apical surface (S2E Fig), which was also F-actin rich (S2F–S2H Fig). Horizontal packing of such rubber string–like fibers by the narrowing of the apical surface seemed to contribute to subapical elasticity. Furthermore, imaging in slice culture revealed a novel subapical-specific (within 5 μm from the surface) cellular structure; lamellipodia-like protrusions were dynamically extended laterally into the surrounding space from almost all apical processes of non–M-phase VZ cells (Fig 3I, S2I Fig and S10 Movie). They were inhibited by blebbistatin (S2J Fig and S11 Movie). The in vivo existence of this novel structure was confirmed by serial block-face scanning electron microscopy (SBF-SEM) (Fig 3J and S12 Movie). The close spatial juxtaposition of this volume-increasing microstructure with the narrowing/contractile apical surface may efficiently increase local subapical elasticity. Together, these results suggest that the subapical space, where basal IKNM (i.e., newly generated daughter cells’ nucleokinesis) begins, is indeed elastic.

### In silico and cell-biological analyses of the M-to-G1 transition support an actomyosin-dependent bouncing-like mechanism of nucleokinesis

**Fig 4:**
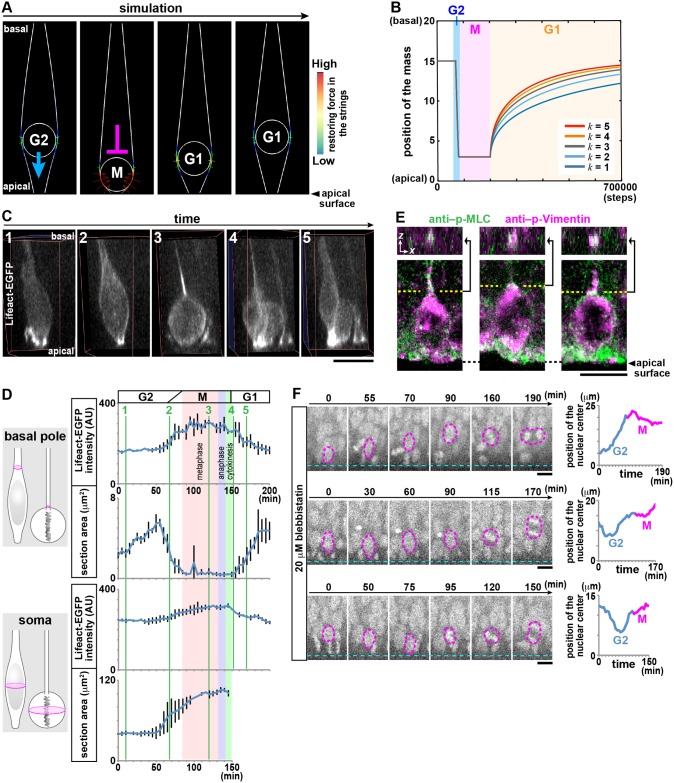
In silico and cell-biological findings support an actomyosin-dependent bouncing-like mechanism for daughter cells’ initial nucleokinesis. (A) Mathematical simulation showing a bouncing-like basal displacement of a G1-phase cell’s nucleus/soma following apical and lateral pushing of the subapical space by the mother cell (G2–M) (see also S13 Movie and S3A Fig). Two strings, fixed to the apical surface, behave elastically, mimicking the densely process-filled subapical space of VZ, with force storage (red in color code [during M phase]) and subsequent release (during early G1). (B) Graph showing the relationship between the spring constant (*k*) of the elastic strings and the trajectory of early G1-phase cells’ nuclei/somata. (C) Three-dimensionally projected view of a clonally Lifeact-EGFP–labeled progenitor cell (panels 1–3) and its daughter cells (panels 4 and 5) (see also S14 Movie). (D) Graphs showing the time-dependent changes in the intensity of Lifeact-EGFP and the horizontal sectional area at the basal pole or the soma of a single progenitor cell from G2 phase to the end of M phase (*n* = 3 clones). Reflecting that most progenitors divide horizontally [39], the horizontal section of the observed cell’s soma became ellipsoidal with the long axis parallel to the apical surface (130–140 min). The thickness of the basal process and the degree of F-actin accumulation (both measured at the portion indicated by the arrowhead) changed in an inversely correlated manner. (E) Accumulation of phospho-myosin-light-chain (green) in the basal process of a phospho-Vimentin^+^ (magenta) M-phase cell, shown in a cross-sectional view and a view obtained by orthogonally sectioning at the dotted line. (F) Myosin II inhibition resulted in basal (up to 20 μm) bouncing of the M-phase cell’s soma (prior to cytokinesis, which occurred at 150–190 min) (see also S3C for in vivo immunohistochemical results and S16 Movie). Scale, 10 μm in C, E, and F. Underlying data can be found in S1 Data. AU, arbitrary unit; p-MLC, phosphorylated myosin light chain; VZ, ventricular zone.

To evaluate our new model, in which elastic energy stored in local subapical tissue is released to generate a force that lifts up daughter cells’ nuclei/somata, we performed both in silico and slice culture analyses, focusing on the origin and redistribution of mechanical forces during the transition from M phase to early G1 phase. In our mechanical simulation, (1) the elastic subapical space was depicted in a simplified manner as two springlike strings fixed at the apical end, and (2) the time-dependent position changes of a single soma (as of G2, M, and early G1 phases) were animated (Fig 4A and 4B, S3A and S3B Fig, S13 Movie). We first applied a constant apicalward force to the center of nucleus/soma in G2 phase and further, until the end of M phase. Reaction forces from the elastic strings generated vertical (basal) components, i.e., a lifting force. Thus, the strings successfully mediated a basal bouncing-like movement of the circle (daughter cell’s nucleus/soma) away from the apical surface through sequential transfers of mechanical energy from each laterally expanding M-phase progenitor cell (providing a pushing force) to (1) the subapical space (storing elastic energy) and (2) back to the nuclei/somata of that progenitor cell’s daughters.

In silico basalward bouncing of the daughter cell’s soma/nucleus occurred following a timely apicalward force from the M-phase mother cell’s round soma, as if it were pushed by a finger (S1B and S1C Fig, S4 Movie), at its basal pole. Because previous studies have suggested that actomyosin localized basal to the nucleus/soma of G2/M-phase cells may contribute to apical nucleokinesis and rounding up [21, 36–38], we speculated that actomyosin provides the cell-biological basis for such transient apicalward pushing (until the end of M phase). To explore this possibility, we live monitored actin dynamics. Interphase cells exhibited F-actin enrichment at the apical junction. During the G2-to-M transition, F-actin accumulated near the basal pole of the soma (Fig 4C and 4D, S14 and S15 Movies), followed immediately by thinning of the basal process and subsequently by rounding up of the soma. F-actin accumulation at the basal side continued during rounding up of the soma and the growth of cleavage furrow in a basal-to-apical direction and then became undetectable after completion of cytokinesis. Immunohistochemistry revealed that the basal F-actin–rich region accumulated phospho-myosin light chain (Fig 4E).

To determine whether the basal myosin functions as the apicalward presser of the soma (i.e., the squeezer of the cytoplasm), we live monitored M-phase cells in cerebral walls treated with blebbistatin (20 μm). Although this treatment caused medium-immersed (unembedded) cerebral walls to freely undergo a concave-to-convex change (Fig 3E), embedding of cerebral wall slices in collagen gel allowed them to fully maintain their original apically concave shape even in the presence of blebbistatin, thereby keeping the subapical space laterally compressed (at least to a considerable degree). We observed abnormal basal bouncing of the blebbistatin-treated M-phase cells’ somata (Fig 4F, S3C Fig, S16 Movie). These data are consistent with our model, in which each M-phase cell’s soma is squeezed from the basal pole during its rounding-up step in an actomyosin-dependent manner and thus laterally pushes the surrounding subapical space.

**Fig 5:**
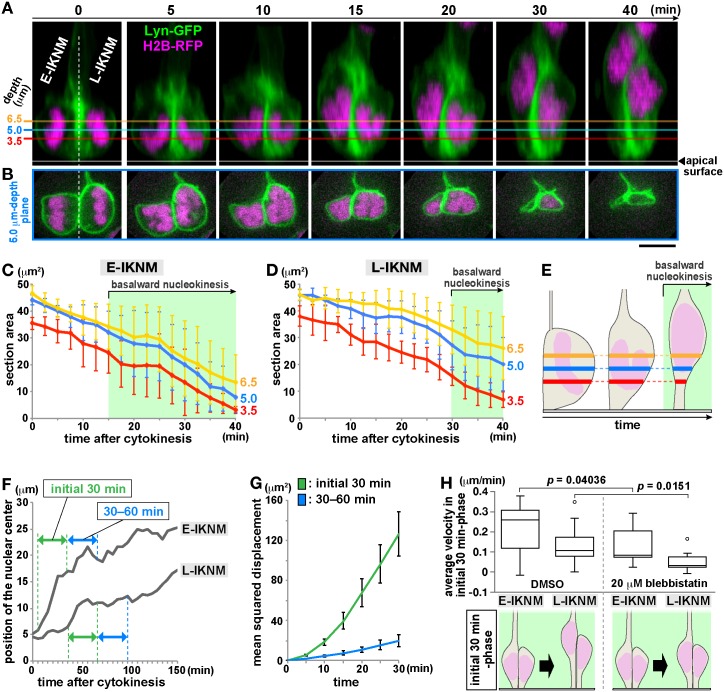
Initial basal IKNMs are preceded by apical cytoplasmic narrowing, quicker than the following basal IKNMs, and assisted by actomyosin. (A–E) Three-dimensionally reconstructed time-lapse observations of a representative pair of sister daughter cells (A, cross sectional; B, horizontal at a depth of 5 μm) used for the measurement of the horizontal sectional area at 3.5 μm, 5.0 μm, and 6.5 μm in the E-IKNM (i.e., basal process-inheriting) daughter (C) and L-IKNM daughters (D) (*n* = 5 pairs), showing that an apical-to-basal cytoplasmic thinning of newborn daughter cells precedes the onset of basal nucleokinesis (E). (F and G) Two different phases in basal nucleokinesis, shown by a graph depicting nuclear/somal displacement (F) and a graph comparing MSD between the initial (within 30-min, green) and subsequent (30–60-min, blue) phases (G, *n* = 6 pairs). (H) Deceleration of the initial phase by inhibition of myosin II activity (see also S3D and S3E Fig) (*n* = 9 pairs for DMSO, 11 pairs for blebbistatin). Scale, 5 μm in A and B. Underlying data can be found in S1 Data. E-IKNM, early-nucleokinetic; IKNM, interkinetic nuclear migration; L-IKNM, late-nucleokinetic; MSD, mean-squared displacement.

Based on previous studies that well described the IKNM dynamics [18–21, 23–26], we extended our analysis on the behaviors of newborn (≤3-hr-old) daughter cells to characterize the relationship between the shapes of their apical cytoplasmic portions and the positions of their nuclei (Fig 5). Because we reasoned that the initial step would be more susceptible to the hypothetical elasticity-based pushing by the subapical space than later steps, it was also important to determine whether basalward nucleokinesis proceeded constantly or, instead, exhibited transitions from a fast mode to a slower mode. Soon after their generation, most daughter cells remain connected to the apical surface [6, 39]. Sister daughter cells’ nuclei depart from the subapical space sequentially; one daughter cell inherits the basal process from its mother cell [5, 6] and initiates basal nucleokinesis more quickly than its sibling (non–process-inheriting) cell [18, 27, 40]. Despite this morphology-dependent intra-clonal difference in nuclear-departure time, we identified two key rules common to the early-nucleokinetic (E-IKNM) and late-nucleokinetic (L-IKNM) daughter cells. First, the reduction of the horizontal sectional area of the apical cytoplasm (at 3.5 μm, 5.0 μm, and 6.5 μm from the apical surface) was earliest at 3.5 μm and later at more basal positions, suggesting an apical-to-basal cytoplasmic thinning, and always preceded the onset of basal nucleokinesis (Fig 5C–5E). Second, basal nucleokinesis, once started, was mostly biphasic, with an initial (within 30-min) quick displacement phase occurring in the subapical space and a subsequent slower phase occurring in more basal regions (Fig 5F and 5G). When myosin II was pharmacologically inhibited with 20 μm blebbistatin, both the initial quicker phase and the subsequent slower phases were decelerated (in both E-IKNM and L-IKNM) (Fig 5H, S3D and S3E Fig).

### Mechanical experiments support an elasticity-based passive displacement of daughter cells’ nuclei/somata from the Windkessel-like subapical space

**Fig 6:**
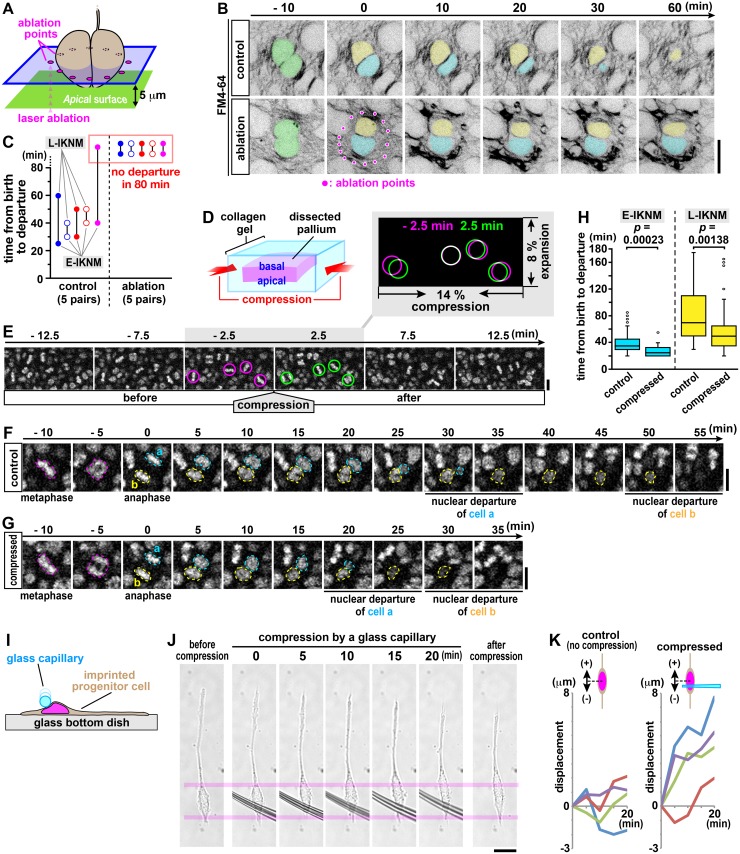
Decompression and compression experiments support the elasticity-based passive nucleokinesis mechanism. (A–C) Artificial shrinkage of the process-rich subapical space by laser ablation (at 5 μm deep, as illustrated in A; circularly darkened in FM4-64 imaging, B; see also S3F Fig and S19 Movie) inhibited the initial basal displacement of daughter cells’ nuclei/somata (*n* = 5 pairs, C) (see also S18 Movie). (D and E) Unidirectional centripetal compression of a cerebral wall (with a device, as shown in D) increased the nuclear density in the subapical space (E; see also S3G Fig and S20 Movie). (F–H) Initial nucleokinesis from the compressed/deformed subapical space (G; H2B-mCherry–labeled) was quicker than that from the uncompressed subapical space (F). Time until departure was 28 ± 8 min for E-IKNM and 60 ± 36 min for L-IKNM when compressed, whereas it was 39 ± 15 min for E-IKNM and 82 ± 39 min for L-IKNM in control (*p* = 0.00023 for E-IKNM and *p* = 0.00138 for L-IKNM, Mann–Whitney *U* test) (summarized in H, *n* = 59 pairs for control and 43 pairs for the compressed condition) (see also S21 and S22 Movies). (I–K) Compression of single “imprinted” progenitor cells. VZ cells attached to dish base, with their spindle or bipolar in vivo–like morphologies, were pressed perinuclearly by a capillary (illustrated in I, pictured in J), and their nuclei/somata underwent counter-directional displacements (exemplified in J [see also S3H Fig], summarized in K, *n* = 4 for both control and compression). Scale, 10 μm in B, E, F, G, and J. Underlying data can be found in S1 Data. E-IKNM, early-nucleokinetic; L-IKNM, late-nucleokinetic; VZ, ventricular zone.

The results described above support non–cell-autonomous basal nucleokinesis driven by a force externally applied from the subapical space, where actomyosin serves to make the entire apical surface contractile (thereby contributing to the subapical elasticity) and to squeeze/push each M-phase cell’s cytoplasm apically from its basal pole (to begin the lateral storage of elastic energy). However, they are also consistent with a cell-autonomous mechanism in which daughter cell–intrinsic actomyosin near the apical surface might transiently squeeze/push daughter cells’ nuclei basally [22]. In silico increase and decrease of the spring constants of the two lines resulted in accelerated and retarded basal nucleokinesis, respectively (Fig 4B). Accordingly, to functionally assess the former (passive) mechanism, we performed a set of mechanical experiments to determine whether external forces, as expected to arise in the elastic (Windkessel-like) subapical space, are necessary and sufficient for the basal nucleokinesis. Specifically, we locally laser ablated the subapical space that surrounded cells immediately (<5 min) after cytokinesis (Fig 6A). Successful ablation of the surrounding space, not the nuclei/somata whose movement should be evaluated, was reflected by tissue irregularity or shrinkage (Fig 6B, S3F Fig, S18 and S19 Movies). Although nuclei of daughter cells in control slices departed from the subapical space in 33 ± 6 min in E-IKNM cases or 58 ± 17 min in L-IKNM cases (*n* = 5 pairs), nuclei of daughter cells whose neighboring subapical space was laser ablated (decompressed) did not move for up to 80 min (*n* = 5 pairs) (Fig 6C).

We then asked whether compression to increase the subapical elasticity would accelerate daughter cells’ initial nucleokinesis. To this end, we placed a cerebral wall slice embedded in collagen gel in a silicone-rubber chamber, to which an external force can be applied along a single axis to provide about 14% compression (and about 8% stretching along the orthogonal axis) of the subapical space (Fig 6D–6H, S3G Fig, S20–S22 Movies). From this artificially compressed subapical space, both E-IKNM daughter cells’ and L-IKNM daughter cells’ nuclei/somata departed significantly earlier than in control cases (Fig 6H).

Next, we sought to determine whether a local external strain, e.g., a strain provided from the subapical space to the apicalmost cytoplasm of a newly generated cell, can actually move that cell’s nucleus/soma against the expected basal mechanical resistance (e.g., intracellular viscosity and/or the extendibility of plasma membrane). To mimic the in vivo situation in a more directly manipulable manner, we “imprinted” VZ tissues onto a culture dish so that the bipolar-shaped geometry of VZ cells could be maintained to a considerable degree [41] (Fig 6I). In imprinted cells successfully pushed by a microcapillary from one pole of the nucleus/soma, nuclear/somal displacement to the opposite side was observed either relatively quickly (within 5 min in 3 out of 4 cases) or slowly (more than 15 min in 1 out of 4 cases), whereas control uncompressed cells did not show such displacements (Fig 6J and 6K, S3H Fig, S1 Data). These results, together with the aforementioned basalward bouncing of M-phase cells’ somata that occurred through overcoming potential apicalward resistance (Fig 4F), imply that (1) external forces are necessary and sufficient for the initial passive movement of nucleus/soma in daughter cells, and (2) the retardation in the initial quick basal nucleokinesis step caused by blebbistatin (Fig 5H and S3E Fig) can be most reasonably explained by the absence or reduction of external forces in the Windkessel-like subapical space, although we do not exclude cell-intrinsic actomyosin’s partial contribution to this initial basalward step.

### Mathematically IKNM-simulated virtual VZ suggests that the initial elasticity-based boosting step is essential for ordered high-degree pseudostratification

**Fig 7:**
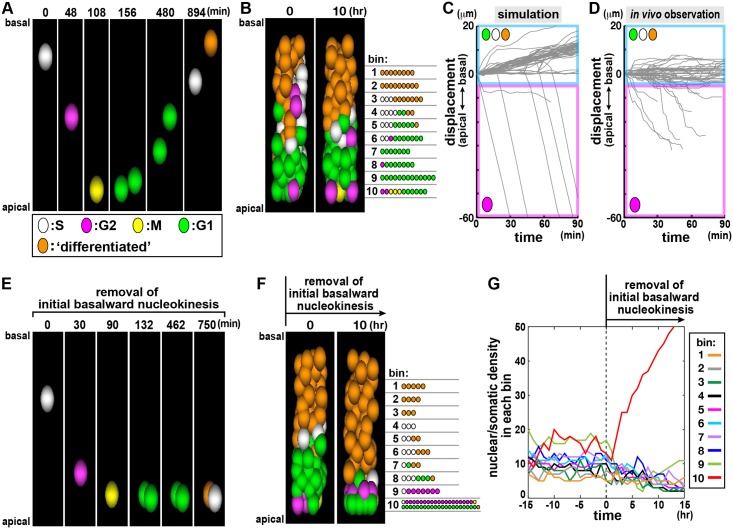
In silico simulation reveals the importance of the initial basalward nucleokinesis for well-organized pseudostratification. (A) Simulation of a single clone from S phase (0 min, white) to M phase (108 min, yellow), and further to the two daughter cells’ basal nucleokinesis (until 894 min, initially green during G1, turning white [entered S phase] and orange [exited from cell cycle = differentiated]) (see also S4A Fig and S23 Movie [left]). (B) Horizontal assembly of single IKNM-simulated clones results in the formation of highly pseudostratified virtual neuroepithelium (see also S23 Movie [right]). (C and D) Graphs comparing the trajectories of nuclei/somata between simulation (C, *n* = 60) and real observation (D, *n* = 60) (see also S4B–S4G Fig). (E–G) In silico removal (from t = 0 min) of the initial basalward propelling force (illustrated in S4A Fig, also visualized in S4G Fig, see Materials and methods for details) resulted in unsuccessful basal displacement of early G1 daughter cells’ nuclei/somata from the subapical space (E) and disruption of the virtual neuroepithelial structure (F and G; nuclear density in bin 10 is abnormally raised). Underlying data can be found in S1 Data. IKNM, interkinetic nuclear migration.

Based on these results, we sought to determine the contribution of the initial elasticity-based boosting step provided by the Windkessel-like subapical space to all IKNMs and the formation of highly pseudostratified NE/VZ. For technical reasons, the aforementioned pharmacological and mechanical perturbations did not permit long-term assessments of neuroepithelia. Hence, we aimed at mathematically removing or inhibiting only the initial 10-μm basalward nucleokinesis phase, to which this Windkessel-like mechanism seemed to critically contribute. We developed a new simulation to systematically describe movements of all VZ cells’ nuclei, individually linked with cell cycle progression (Fig 7A–7D, S4A Fig, S23 Movie), based on previously reported parameters for cell cycle progression and cell differentiation [3, 42] as well as nuclear densities and migration velocities obtained from live-tracked nuclei [18] in the mid-embryonic mouse cerebral VZ.

The two major physical forces applied to induce nuclear/somal displacements along the apicobasal axis were (1) direct soma–soma (nuclear–nuclear) interactions (i.e., collisions and repulsions) and (2) mechanical forces that act in a cell cycle phase–dependent manner, independently of such direct soma–soma repulsions (S4A Fig). The latter (designated as “non-collision” forces) included both intracellular forces dependent on microtubules or actomyosin and external forces such as those provided elastically from the cellular processes to the nuclei/somata. For example, the in silico G2-phase cells’ rapid (highly directional) apical displacement [18–21, 23–26] was reproduced by application of the “non-collision” force. Horizontal assembly/bundling of the nuclear/somal movements of individually simulated clonally related cells (exemplified in Fig 7A, S4A Fig, S23 Movie [left], showing hairpin loop–like, distally bifurcated “production lines”) successfully established a virtual (NE/VZ-like) pseudostratified tissue (Fig 7B, S23 Movie [right]), which recapitulated the overall IKNM trajectories in vivo (Fig 7C and 7D, S4B and S4C Fig). Consistent with real observations (Fig 2B–2E), soma–soma collisions/repulsions were much less frequent in the subapical (within10 μm) part than in more basal parts of the virtual VZ (S4D Fig).

Interestingly, basal nucleokinesis in this simulation was functionally biphasic (S4E–S4G Fig, S24 Movie), reminiscent of real observations (Fig 5F and 5G). The simulations showed that G1-phase nuclei/somata existing >10 μm away from the apical surface could be automatically displaced through direct nucleus–nucleus repulsions (probably between G1-phase cells) alone, as long as movements of other chronologically different nuclei (i.e., apical arrival of G2-phase cells’ nuclei/somata and the initial basal displacement of early G1-phase cells’ nuclei/somata in the subapical [within 10 μm] space) were secured (S4B–S4G Fig), supporting the previously proposed idea of passive IKNM [9, 21, 25, 26]. By contrast, the subapical (within 10 μm) nuclei/somata strongly required a basal acceleration by the “non-collision” mechanism, the absence of which resulted in severe disruption of overall IKNM and the VZ structure (Fig 7E–7G, S25 Movie), suggesting that this initial basalward step may be critical or rate limiting for a high degree of overall pseudostratification. As we experimentally demonstrated in Fig 6, the initial 10-μm basalward step may strongly depend on an external, elasticity-dependent mechanism. Therefore, it is very likely that the initial basal nucleokinesis step, which is more rapid and directional than the subsequent (more basal) nucleokinesis (Fig 5) and requires external elasticity, is critical for the ordered brain histogenesis.

## Discussion

**Fig 8:**
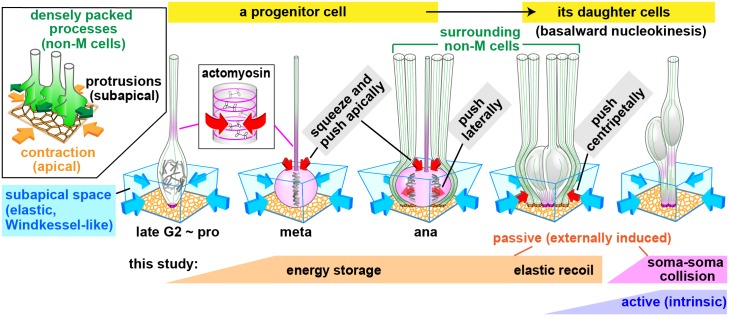
Scheme of elasticity-based passive nucleokinesis of newborn daughter cells. Progression of mitosis through actomyosin-based narrowing of the basal process causes lateral expansion/pushing by the M-phase cell’s soma (red arrow) and deformation of the subapical space that is elastic (blue arrow). Newborn daughter cells’ basalward nucleokinesis is assisted by a restoring force (red arrow) of the deformed subapical space (see also S4H Fig).

We found that NPCs in the highly pseudostratified proliferative zone of the developing mammalian brain undergo elasticity-based passive nucleokinesis from the Windkessel-like subapical space. In this mechanism, tightly neighboring M-phase cells and non–M-phase cells collaborate to efficiently generate local tissue elasticity and also store and provide mechanical energy (Fig 8), displaying a surprisingly mutualistic relationship, in order to safely and economically maintain a large-scale, 3D cell-production system (S4H Fig). This tissue-forming efficiency is reminiscent of minimizing the total energetic expenditure in birds in V formation (which fly alternatingly either as a leader, to generate wing-tip vortices, or as the followers, to utilize a lifting force by the leader-driven vortices) [43].

### “Mechanical mutualism” between M-phase cells and non–M-phase cells underlies a system-level brain-forming order

In diverse proliferative cell types, each non–M-phase cell will eventually enter M phase, and the M-phase cell will then generate two non–M-phase (daughter) cells. Non–M-phase cells and M-phase cells are therefore in a producer–product relationship. This chronological (cell cycle) partnership depends on intracellular chemical reactions, such as the activation and disappearance of cyclins, but a recent study on the *Drosophila* epithelium showed M-phase cells’ nonchemical (physical) contribution to tissue morphogenesis [44].

Following the previously established concept of a mother-to-daughter morphological gift in the developing mouse cortical VZ, i.e., asymmetric inheritance of each M-phase cell’s basal process/fiber by one daughter cell [6, 18], the present study revealed that each M-phase cell also gives mechanical energy to both daughter cells, with elastic assistance from the densely packed apical processes of neighboring non–M-phase cells (via contractility of the apical surface) (Fig 8) and other M-phase cells that divide (to generally increase the pressure of the subapical space) (S4H Fig). These mother-to-daughter (intra-clonal) physical gifts assist daughter cells’ prompt nucleosomal movement away from the subapical space. Thus, such established initial basal nucleokinesis enables non–M-phase cells to have thin and flexible apical processes throughout the subapical space, which is permissive for the voluminous division of new M-phase cells. This permissiveness can be regarded as a mechanical gift from non–M-phase cells to M-phase cells. These bidirectional physical collaborations (S4H Fig) may underlie efficient and safe intra-neuroepithelial nuclear/somal logistics and protect the subapical space from local overcrowding. Existence of too many nuclei/somata in the subapical space prevents progenitor cells from freely dividing at the apical surface and induces them to abnormally leave the apical surface, leading to heterotopic divisions and disruption of histogenesis [18]. Thus, give-and-take relationships (i.e., mutualism) exhibited spatially and physically between M-phase cells and non–M-phase cells (S4H Fig) are essential for ordered brain development.

### Relationship between the active nucleokinesis and the passive nucleokinesis

Previous studies showed that the basal nucleokinesis is mediated by active intracellular mechanisms dependent on kinesin/microtubules [23] and actomyosin [22]. Our IKNM simulation (virtual VZ) (Fig 7) showed that collective basalward IKNMs at basal VZ levels (more than 10 μm from the apical surface) can occur almost passively using soma–soma collisions between G1-phase cells’ nuclei/somata as a major driving force. Although this result is consistent with a model of the passive basal IKNM suggested by Norden et al. [21, 25] and Kosodo et al. [26], the present study cannot address the relative importance of the collision-based passive mechanism compared to the cell-intrinsic (kinesin- and/or actomyosin-dependent) basal nucleokinesis mechanisms.

In the subapical space (within 10 μm from the apical surface), we found a novel passive IKNM mechanism mediated by tissue elasticity and indirect energy transfer. We cannot precisely determine the relative importance of this boosting mechanism compared to the kinesin- and/or actomyosin-dependent mechanisms in the subapical space. Nevertheless, the existence of the Windkessel-like (elasticity-based) boosting mechanism is strongly suggested based on (1) the elastic property of the subapical space (Fig 3), (2) the more directional displacement of nuclei/somata during the initial 30 min than later periods (with more superlinear MSD curves) (Fig 5), and (3) the decompression and compression experiments that resulted in deceleration and acceleration of initial basal nucleokinesis, respectively (Fig 6).

We speculate that a Windkessel-like boosting mechanism may collaborate with cell-intrinsic basal nucleokinesis mechanisms. For example, centripetal mechanical stimuli applied externally from the subapical space might also trigger intracellular molecular machinery for active nucleokinesis, a possibility to be studied in the future. Similarly, cell cycle progression, which is associated with (or upstream to) intracellular molecular mechanisms for nucleokinesis, might also be modulated by external mechanical stimuli—another question to be addressed for better system-level understanding of intra-VZ collective behaviors of NPCs.

### Elasticity as an energy-efficient device for “production logistics” and a parameter for system-level understanding of VZ in development and evolution

Atomic force microscopy (AFM) revealed that the elastic modulus on/near the apical surface was much greater (1,400 Pa) [45] than that in more basal VZ regions (about 100 Pa) [46]. The present study showed that an elasticity-based mechanism assists in an initial basalward step in IKNMs. Actually, diverse biological systems for coordinating heterogeneous movements or flows similarly utilize elasticity as a means to minimize total energetic expenditure. In mammalian running [47, 48] or insect flying [49], elastic energy stored at one stage in the stride or wingbeat is released at another. Likewise, in blood circulation, the aorta flexibly receives blood ejected from the heart and elastically recoils to forward it (Windkessel effect [28]). Thus, such a commonly used strategy might participate in multiple aspects or phases in the VZ growth or the overall brain development, and perhaps in the neocortical evolution.

We previously showed that elasticity (stiffness) measured on/near the apical surface of the neocortical VZ by AFM was greater in ferrets than in mice [45]. We also found in slice culture that NPCs’ initial basal nuclear/somal movement was quicker in ferrets than in mice [27]. It is therefore possible that elasticity in the subapical space participates in the differential IKNM behaviors between mice and ferret. Our mathematically IKNM-simulated virtual VZ showed that the initial basalward nucleokinesis step is important for the overall high-degree pseudostratification (Fig 7). The degree of pseudostratification (i.e., the thickness of NE/VZ) increases from mice to monkeys [13] and even further in humans [14, 15]. It would be meaningful to study the possible contribution of tissue-level or cell-level elasticity to the thickening of VZ during evolution. Our AFM revealed that single dissociated NPCs are stiffer in mice than in ferrets [45]. Adding such measurable parameters more into our virtual VZ method would be effective to improve our simulation and to expand its feasibility in species other than mice.

## Materials and methods

### Animals

The animal experiments were conducted according to Japanese Act on Welfare and Management of Animals, Guidelines for Proper Conduct of Animal Experiments (published by Science Council of Japan), and Fundamental Guidelines for Proper Conduct of Animal Experiment and Related Activities in Academic Research Institutions (published by Ministry of Education, Culture, Sports, Science and Technology, Japan). All protocols for animal experiments were approved by the Animal Care and Use Committee of Nagoya University (No. 29006). R26-Lyn-Venus transgenic mice (accession No. CDB0254K), R26-H2B-mCherry transgenic mice (accession No. CDB0239K) [18, 50], and R26-ZO1-EGFP transgenic mice (accession No. CDB0260K) [45, 51] were provided by Toshihiko Fujimori (NIBB, Japan). Pregnant ICR mice were obtained from SLC. Embryos at the mid-embryonic stage (embryonic day [E] 13 ± 1) were used.

### Live observation of NPCs and cerebral wall

#### Electroporation of plasmid vectors

Sporadic visualization of NPCs in the VZ of cerebral cortical walls was achieved by transfection with a mixture of pEFX-LPL-LynN-EGFP (0.3 μg/μL), pBA-LPL-H2B-mRFP1 (0.3 μg/μL), pEFX-LPL-EGFP (0.3 μg/μL), pEFX-LPL-LynN-mCherry (0.3 μg/μL), and pEFX-Cre (0.001 μg/μL) by in utero electroporation, as described previously [18]. For live visualization of F-actin in NPCs, a mixture of pEFX-Lifeact-EGFP (0.2 μg/μL) and pEFX-Cre (0.001 μg/μL) was electroporated. For simultaneous visualization of the nucleus/chromosome or apical processes together with the apical surface mesh, a mixture of pBA-LPL-H2B-mRFP1 (0.3 μg/μL) and pEFX-Cre (0.001 μg/μL) or pEFX-LPL-LynN-EGFP (0.1 μg/μL) and pEFX-Cre (0.001 μg/μL) was introduced into R26-ZO1-EGFP transgenic mice.

#### Live imaging

Cross-sectional or en face cultures of cerebral walls were prepared as described previously [6, 18, 52]. Briefly, cerebral walls were microsurgically processed and embedded in a polystyrene cell-culture dish (Corning) with Atelocell IAC-30 collagen gel (Koken) at a concentration of 0.3 mg/mL. Confocal time-lapse images were obtained on a BX51W1 microscope (Olympus) equipped with a CSU-X1 laser scanning confocal unit (Yokogawa) with a 40× objective lens (LUMPLFLN40XW, Olympus) or 100× objective lens (LUMPLFL100XW, Olympus) and an iXon+ EMCCD camera (Andor), in an on-stage culture chamber (Tokai Hit) filled with 45% N_2_, 40% O_2_, and 5% CO_2_. For confocal imaging of all VZ cells in the subapical region (Fig 2B), processed cerebral hemispheric walls were stained with FM4-64 (Thermo Fisher Scientific) at a concentration of 5 μg/mL, as described previously [27, 52]. The areal occupancy of either somata (Fig 2B, defined such that the diameter of each sectional area was >3 μm) or processes was examined as described previously [45].

### Quantitative analyses for nuclear migration and Lifeact-EGFP distribution

Image processing, nuclear tracking, and calculation of cross-sectional area and fluorescent intensity were performed using ImageJ. Fluorescent intensity of Lifeact-EGFP at the level of the soma or the basal pole (as depicted in Fig 4C and 4D) was determined by measuring the maximal pixel intensity (i.e., brightest spots or bands) on the horizontally sectioned cell cortex. Reconstructed fluorescent 3D images were obtained using Volocity (PerkinElmer). “Departure” of each newborn daughter cell’s nucleus/soma from the subapical space under en face observation (as discussed in Fig 6) was defined in the horizontal sectional plane 5 μm from the apical surface as the time when its diameter became smaller than 3 μm (including complete disappearance). Onset of basal displacement of the nucleus/soma of a newborn daughter cell under cross-sectional observation (as discussed in Fig 5, S3D and S3E Fig) was defined as the time when the mass center of the nucleus/soma moved 1.5 μm basally. MSD was obtained based on time-dependent changes of nuclear position along the apicobasal axis, as described previously [18].

### Quantification for the apical surface’s dynamics

To determine whether the apical surface was stable, without exhibiting local tilting or floppiness of apices even where mitosis occurred, we analyzed a 3-min-interval movie obtained by en face observation of the apical surface of a cerebral wall prepared from a R26-ZO1-EGFP transgenic mouse [51] at 0.5-μm *z* intervals. Cell borders at the apical surface level were extracted from the zero-crossing points for first-order derivatives obtained by applying 1D Savitzky-Golay filter along the *x*, *y*, and *z* directions. Peak voxels marked by the filter were then smoothened by morphological operations and skeletonized in 3D space [53]. Individual cell contours were determined by applying modified Dijkstra’s algorithm, which finds the shortest set of edges that represents a distinct cell contour (S2A Fig, green trajectories in the left panels). Normal vectors representing the inclination of each cell apex were estimated by averaging normals for every possible triangular face defined by two neighboring vertices and another vertex in the cell. Angular correlations between the normals of adjacent time points were used to quantify the flatness of the cortical plane (apical surface) (S2A Fig, right panel).

### Slice-bending assay

As previously described [18], coronal cerebral wall slices (250–300 μm thick) were prepared from E12 mouse embryos and then placed into a polystyrene suspension cell culture dish (Corning). After incubation for 30 min with culture medium containing 1% DMSO (Sigma-Aldrich) only or 1% DMSO plus 20 μm blebbistatin (Calbiochem) (Fig 3E), slices were fixed with 4% paraformaldehyde prepared in phosphate buffer (pH 7.4) and then immunostained with anti-ZO1 mouse monoclonal antibody (33–9100, Invitrogen, 1/500), followed by Alexa Fluor 488-labeled anti-mouse IgG antibody (A11029, Thermo Fisher Scientific, 1/1,000). Images were acquired on an MZFLIII fluorescent stereomicroscope (Leica) equipped with an ORCA-ER digital camera (Hamamatsu Photonics).

### Slice culture under blebbistatin treatment

To examine the effect of blebbistatin on VZ cells’ nuclear movements (as shown in Fig 4F and S3C Fig [rebounding of M-phase cells’ somata] and S3D and S3E Fig [reduced initial basal nucleokinesis]) and on the dynamics of the lamellipodia-like protrusions (as shown in S2J Fig), coronal cerebral wall slices (250–300 μm thick) prepared from E13 mouse embryos were embedded in collagen gel prior to incubation in culture medium containing 1% DMSO only or 1% DMSO plus 20 μm blebbistatin. In this collagen-embedded condition, slices, even those treated with blebbistatin, maintained their original apical concavity (Fig 4F and S3C Fig) and density of apical endfeet [45]. Consistent with a previous report [54], progenitor cells in slices treated with 20 μm blebbistatin exhibited cytokinesis.

### Laser ablation experiments

Apices of NPCs were visualized by electroporation with pCAG-EGFP-ZO1 (gift from F. Matsuzaki) [39] at a concentration of 0.5 μg/μL. Cerebral walls were mounted with the apical side down in collagen gel in a glass-bottomed dish (Iwaki) using collagen gel. Laser ablation was performed using a confocal laser-scanning microscope (FV1000 with 15 mW LD473, Olympus) equipped with a UV laser system (UV-ASU-P2, Olympus) and a 60× objective lens (UPLSAPO60XO, Olympus), as described previously [44]. A single pulse of a 349-nm laser illumination was applied to a vertex of cell boundaries simultaneously with the image acquisition [18].

For the ablation of M-phase somata (Fig 3F–3H, S2B–S2D Fig) and apical processes around pairs of newborn daughter cells (Fig 6A–6C), cells were visualized by staining with FM4-64 for 30 min (Figs 3F and 6A–6C); by electroporation with pEFX-LPL-LynN-mCherry and pEFX-Cre (Fig 3G and 3H, S2B and S2C Fig); or by electroporation with pEFX-LPL-LynN-mCherry, pEFX-LPL-EGFP, and pEFX-Cre (S2D Fig). Laser ablation was performed as described previously [55] on an IX81 inverted microscope (Olympus) equipped with CSU-X1 (Yokogawa), iXon3 897 EMCCD camera (Andor), 60× objective lens (UPLSAPO60XW, Olympus), on-stage culture chamber (Tokai Hit) filled with 95% O_2_ and 5% CO_2_, and MicroPoint (Andor), operated with the iQ2 live cell imaging software (Andor). A pulse of 365-nm laser illumination at 16 Hz was simultaneously applied to the center of the soma (Fig 3F–3H, S2B–S2D Fig) and at 15 points in a circle around the newborn daughter cells (Fig 6A–6C) at a horizontal plane 5 μm from the apical surface. Images were acquired at 1-s intervals (Fig 3F–3H), 5-s intervals (S2D Fig), 5-s intervals at the ablation and then 5-min intervals (Fig 6A–6C), or 10-s intervals with z-acquisition (S2B and S2C Fig). In spite of the instantaneous fading of fluorescence, any fragmentation of plasma membrane or cytoplasm was not observed after somal ablation in our experimental condition (Fig 3G, S2B–S2D Fig). On the other hand, the LynN-mCherry–labeled plasma membrane protruded from the apical surface of VZ (S2B and S2C Fig, S26 Movie), suggesting a possibility that the energy from the laser reduced the stability (or increased the fluidity) of the cytoplasm. Although the exact reaction of laser-ablated cytoplasm remains unknown, our observations suggested that the ablated cells immediately lost the original physical stability without losing the continuity of the plasma membrane, thereby allowing the cell to passively take a shrinking geometry according to external compressive force in the subapical space.

### Compression of cerebral walls

A STB-CH-04 silicone chamber (dimensions; 20 × 20 × 10 mm, Strex) was coated overnight at room temperature with 0.1% polyethyleneimine (Sigma-Aldrich) in 150 mM sodium tetraborate (pH 8.4). The chamber was then unidirectionally stretched about 20% using an STB-100 manual-operated stretcher (Strex). In the pre-stretched chamber, a dissected cerebral hemispheric wall (500 × 500 μm) was mounted in 1 mL of collagen gel, with the apical side down. After gel solidification and addition of medium, the chamber was carefully relaxed to the original shape, thereby compressing the gel-embedded cerebral wall. To compare the subapical space before and after compression, confocal microscopic images were obtained in 5-min intervals at 5 μm from the apical surface on an FV1000 laser scanning confocal microscope (Olympus) equipped with a 40× objective lens (UPLFLN40X, Olympus) and an on-stage culture chamber (Tokai Hit) filled with 95% O_2_ and 5% CO_2_.

### Compression of single “imprinted” VZ cells

Bipolar-shaped VZ cells on cell culture dishes were obtained by the cortical imprint method [41], with partial modifications. Briefly, coronal cerebral wall slices (250–300 μm thick) prepared from E13 mouse embryos were incubated for 10 min at 37 °C in phosphate buffer (pH 7.4) containing 10 U/mL papain (Nacalai Tesque) and 100 μg/mL DNase I (Sigma-Aldrich). Slices were then placed onto a glass-bottomed dish (Iwaki) coated with Cell-Tak (Corning) at a concentration of 10 μg/cm^2^ with a minimal volume of growth medium. After incubation for 20 h, slices were gently moved away from the bottom glass by application of excess growth medium and gentle pipetting. For compression of the soma of VZ cells, sharp microneedles were generated from a GD-1 glass capillary with filament (Narishige) using a PN-31 puller (Narishige). To avoid direct association with the bottom glass, the tip of the microneedle was slightly bent using an MF-900 Microforge (Narishige). Compression assay was carried out on an IX71 inverted microscope (Olympus) equipped with an MMO-202ND three-axis joystick oil hydraulic micromanipulator (Narishige), an ORCA-ER digital camera (Hamamatsu Photonics), and an on-stage heater (Tokai Hit). The off-center surface of the soma was gently compressed with the lateral part of the microneedle, held in an HI-7 injection holder (Narishige). The indentation depth was ≤3 μm, as estimated by the *z* axis scale on the micromanipulator. After compression and image acquisition, VZ cells were immediately fixed and immunostained with anti-Sox2 antibody (ab97959, Abcam, 1/500).

### Immunohistochemistry

Cross-sectional and en face immunohistochemistry were performed as described previously [18, 56], with partial modifications. For immunostaining of phospho-myosin light chain and phospho-vimentin, embryonic brains were fixed for 1 h at 4 °C with periodate-lysine-paraformaldehyde (PLP) fixative containing 2% trichloroacetic acid. For immunostaining of microtubules, brains were fixed for 1 h at room temperature by 4% paraformaldehyde fixative containing 1 μm Taxol and 0.1% Triton X-100. Frozen sections (16 μm thick) were treated with the following primary antibodies: anti–α-tubulin (T6199, Sigma-Aldrich, 1/1,000), anti–phospho-myosin light chain (ab2480, Abcam, 1/500), and anti–phospho-vimentin (D076-3, MBL, 1/500). Sections were then treated with secondary antibodies conjugated with Alexa Fluor 488 or 546 (A11029, A11034, A10036, Thermo Fisher Scientific, 1/1,000) or Alexa Fluor 546–conjugated phalloidin (A22283, Thermo Fisher Scientific, 1/1,000). Confocal images were obtained on an FV1000 laser scanning confocal microscope (Olympus).

### Electron microscopy

Scanning electron microscopy of cerebral hemispheres was performed as described previously [45]. In brief, cerebral hemispheres were fixed in 4% paraformaldehyde in phosphate buffer (pH 7.4) and subsequently in 2.5% glutaraldehyde in phosphate buffer (pH 7.4). The brains were further postfixed at 4 °C overnight in 2.5% glutaraldehyde, followed by incubation with 1% osmium tetroxide in phosphate buffer (pH 7.4) and dehydration. The samples were trimmed, coated with carbon, and examined under an S-800S SEM (Hitachi).

For SBF-SEM, cerebral hemispheres obtained from E13 mouse embryos were fixed for 3 h at 4 °C in PLP fixative containing 2.5% glutaraldehyde. Tissue processing for SBF-SEM was performed as described previously [57] with partial modifications. En bloc heavy metal staining was carried out for 1 h in 2% osmium tetroxide solution containing 150 mM sodium cacodylate, 2 mM calcium chloride, and 1.5% potassium ferrocyanide. Hemispheres were then treated with 1% thiocarbohydrazide for 20 min at room temperature, 2% osmium tetroxide for 30 min at room temperature, and 1% uranyl acetate overnight at 4 °C, followed by lead aspartate staining. After dehydration and embedding in Durcupan ACM (Sigma-Aldrich), serial images of the subapical space in the VZ were collected on a scanning electron microscope (Marlin, Zeiss) equipped with a 3-View ultramicrotome (Gatan); *z* step was 50 nm and acceleration voltage was 1.4–2.0 kV. Images were captured at 4,730× magnification in a field of 8,192 × 8,192 (*x* and *y* axes). Captured images were aligned and reconstructed using the Reconstruct software [58] (available at SynapseWeb, http://synapseweb.clm.utexas.edu/).

### Mathematical simulation for bouncing of the nucleus/soma from the elastic subapical space

To simulate the mechanical behaviors of the nucleus/soma of G2/M-phase or early G1-phase cells in the elastic subapical space, the translocation of the nucleus/soma in question and the potential centripetal mechanical influences from the elastic subapical space to the nucleus/soma were simulated in a cross section containing the apicobasal axis (Fig 4A and S3A Fig), assuming rotational symmetry of the nucleus/soma around the apicobasal axis. The subapical space, which is densely filled with cellular processes and other cells’ nuclei/somata, was represented as two effective elastic strings that tightly sandwiched and held the nucleus/soma. The motion of the center of mass of the nucleus/soma with position vector **R** at time *t* can be described by the following equation:

$$\Gamma \frac{dR}{dt}=-\frac{\partial U_{rep}}{\partial R}+F_{act}\;.$$ (1)

Here, Γ is the friction coefficient. The right-hand side of Eq 1 represents the force exerted on the center of mass of the nucleus/soma; the first term is derived from the repulsive energy function *U*_rep_, which represents the volume-exclusion effect of the nucleus/soma against the strings, and the second term, **F**_act_, is a force vector representing the driving force of apicalward translocation in the period from G2 to M phase.

The elastic string is described as *N*+1 particles connected by *N* massless springs. The motion of particle *i* with position vector **r**_*i*_ can be described by

$$\gamma \frac{dr_{i}}{dt}=-\frac{\partial }{\partial r_{i}}(U_{els}+U_{rep})\;.$$ (2)

Here, γ is the friction coefficient. The right-hand side of Eq 2, representing the force exerted on the particle, is derived from energy functions. The elastic energy stored in the string, *U*_els_, is expressed by a harmonic potential energy calculated from the positions of each particle:

$$U_{els}=\sum_{i}{\frac{k}{2}(|r_{i+1}-r_{i}|-l_{0})}^{2}\;,$$ (3)

where *k* is the spring constant and *l*_0_ is the spring length in the stress-free configuration.

The volume-exclusion effect of the nucleus/soma is introduced by a repulsive interaction between the particle *i* and the nucleus/soma. Assuming that the shape of the nucleus/soma is well approximated by a sphere (circle in the cross section) of radius σ, the repulsive potential, *U*_rep_, is expressed by

$$U_{rep}=\sum_{i}\varepsilon {\left(\frac{\sigma }{\left|r_{i}-R\right|}\right)}^{12}\;,$$ (4)

where, ε (>0) is the repulsion energy constant.

All model constants are listed in Table 1. Here, to focus on the bouncing-like basal displacement, both ends of the strings are fixed as the boundary condition. The lateral distance between the fixed ends of the two strings, and the apicobasal distance between the fixed ends of the strings, are represented by *d* and *L*, respectively (S3A Fig).

**Table 1:** Model constants.

Symbol	Value	Description
***k***	3.0	Spring constant in Eq 3
***l*_0_**	1.0	Spring length at stress-free state in Eq 3
***N***	30	The number of springs comprising the string
**ε**	1.0 × 10^−2^	Repulsive energy constant in Eq 4
***σ***	3.0	Radius of the nucleus/soma in Eq 4
**F_act_**	(0, −0.15)	Apicalward force vector from G2 to M phases in Eq 1
**Γ**	1.0	Friction coefficient of the nucleus/soma in Eq 1
**γ**	1.0 × 10^−1^	Friction coefficient of particle in Eq 2
***d***	2.0	Lateral distance between the fixed ends of the two strings
***L***	3.0 × 10^1^	Apicobasal distance between the fixed ends of the strings

### Mathematical simulation for a virtual VZ to reproduce all cells’ IKNMs

We formulated a particle-based mathematical model to reproduce the movements of all VZ cells’ nuclei/somata (Fig 7A–7G and S4A Fig), which is similar to Kosodo et al. [26]. Parameters were set based on our live observation of all VZ cells’ nuclei [18]. At first, we defined the center of the *i*th nuclei as ***r***_*i*_ (note: each bold character represents a 3D vector). Next, we defined the movement of the nucleus/soma by the following governing equation:

$$\frac{dr_{i}}{dt}=\sum_{j=1}^{N}f(\frac{r_{j}-r_{i}}{\left|r_{j}-r_{i}\right|})+g(\phi_{i})+h(R_{i}-r_{i}).$$ (5)

The left-hand side $\frac{dr_{i}}{dt}$ represents the change in position of the *i*th nucleus per unit time.

The first term of the right-hand side $\sum_{j=1}^{N}f(\frac{r_{j}-r_{i}}{\left|r_{j}-r_{i}\right|})$ represents the interaction between nuclei/somata. We assumed that nuclei/somata within a certain distance could repel each other, yielding function ***f*** as follows:

$$f\left(r\right)=\{\begin{array}{c} -\beta \left(\alpha -\left|r\right|\right)\frac{r}{\left|r\right|}\;\left(\left|r\right|\leq \alpha \right)\\ 0\;(\left|r\right|>\alpha ) \end{array}.$$ (6)

Here, *α* represents the size of the nucleus/soma and *β* represents the stiffness of the nucleus/soma. Note that basal nuclear/somal displacements by $\sum_{j=1}^{N}f(\frac{r_{j}-r_{i}}{\left|r_{j}-r_{i}\right|})$ in Eq 5 was minimal at *z* < 10 (during the initial 10-μm step) (S4D Fig), consistent with real observations in slice culture (Fig 2B–2E).

The second term of the right-hand side **g**(*ϕ*_*i*_) represents the force we applied in the *z* direction (either apically or basally) according to a developmental clock (mostly corresponding to the progression of the cell cycle, with two exceptions described below). *ϕ*_*i*_, the time or phase of a given VZ cell’s nucleus/soma, was defined as follows: *ϕ* = 1, G2 phase (1 h); *ϕ* = 2, M phase (1 h); *ϕ* = 3, G1 phase (9 ± 1 h) of a daughter cell that has inherited the basal process from its mother cell progresses (i.e., departs the subapical space earlier than its sibling cell’s nucleus/soma, “early-nucleokinetic” [E-IKNM]); *ϕ* = 3′, G1 phase of a daughter cell that has not inherited the basal process from its mother cell progresses (i.e., departs the subapical space later than its sibling cell’s nucleus/soma, “late-nucleokinetic” [L-IKNM]); *ϕ* = 4, S phase (4 h); and *ϕ* = 5, a VZ cell that has progressed through G1 phase and then decided not to enter S phase (cell cycle exited, “differentiated”) to subsequently leave the VZ. The duration assigned to each cell cycle phase was based on values previously estimated in vivo by Takahashi et al. [3]. According to Takahashi et al. [42], the probability that a given G1-phase cell enters S phase (*ϕ* = 4) was set to 67%, whereas the probability of differentiation (*ϕ* = 5) was 33%. To reproduce the sequential nuclear/somal departure of sister cells (i.e., E-IKNM cells and L-IKNM cells) [18], a lag time (0–3 h, random) was set between *ϕ* = 2 and *ϕ* = 3′, whereas *ϕ* = 2 and *ϕ* = 3 were directly connected (S4A Fig). Although the cell cycle would progress continuously from the end of M phase even in the L-IKNM daughter cells, our IKNM simulation described the sequential departures through a technical delay in cell cycle initiation (S4A Fig). The propelling force can be defined as follows:

$$g\left(\phi \right)=-V\left(\phi \right)\;Z_{1}\;v.$$ (7)

**Z**_**1**_ = (0,0,1) represents a unit vector toward the *z* direction and *v* the velocity of the nucleus/soma. *V*(*ϕ*) represents the force that was applied according to the cell cycle phase of each VZ cell (the owner of the nucleus/soma), defined as follows:

$$V\left(\phi \right)=\{\begin{array}{c} 8.5\;\left(\phi =1\right)\\ \begin{array}{c} 0\;\left(\phi =2\right)\\ -1.6\;\left(\phi =3\;and\;z\;<10\right)\\ 0\;\left(\phi =3\;and\;z\;\geq 10\right)\\ -1.6\;\left(\phi =3\prime \;and\;z\;<\;10\;\right)\\ 0\;\left(\phi =3^{\prime }\;and\;z\;\geq \;10\;\right) \end{array}\\ 0\;\left(\phi =4\right)\\ 0\;\left(\phi =5\right) \end{array}.$$ (8)

Although basal nucleokinesis is mediated by active intracellular mechanisms dependent on kinesin/microtubules [23] and actomyosin [22], our IKNM simulation showed that collective basalward IKNM at *z* ≥ 10(VZ levels more than 10 μm from the apical surface) can occur almost passively (i.e., without the second term, depending only on the first term in Eq 5), consistent with a model suggested by Norden et al. [21, 25] and Kosodo et al. [26]. This passive in silico nuclear/somal movement at *z* ≥ 10 was possible, however, only when a basalward force was applied during *z* < 10 (during the initial 10-μm step) to both the E-IKNM (*ϕ* = 3) and L-IKNM (*ϕ* = 3′) daughter cells using the second term. The density of nuclei/soma in this *z* < 10 zone is low; therefore, the contribution of the first term is very small. The second term at *z* < 10 does not distinguish the intracellular mechanisms [22, 23] from the elasticity-based (Windkessel-like) passive mechanism that we propose.

The third term of the right-hand side **h**(***R***_*i*_ − ***r***_*i*_) represents lateral constraints to the nucleus/soma. This term was set based on our observation that nuclei/somata of VZ cells do not freely move laterally beyond 3 μm [18]. Although not shown in the animation, we required that apical and basal processes attach to the apical and basal surfaces of the virtual cerebral wall. ***R***_*i*_ = (*x*_*i*_, *y*_*i*_, 0) represents the positions where these processes were attached to the apical and basal surfaces. **h**(***R***_*i*_ − ***r***_*i*_) is defined as follows:

$$h\left(R_{i}-r_{i}\right)=\gamma \left(R_{i}-r_{i}\right)\cdot Z_{2}.$$ (9)

***Z***_**2**_ = (1,1,0) is a vector used to limit the movement by structural constraint within the *xy*-plane, and *γ* represents the strength of the constraint. Parameters were set as follows: *α* = 10, *β* = 15, *γ* = 4.

At the starting point of the simulation, we assigned 96 nuclei/somata (10 in G2 phase and 86 in G1 phase) in 20 × 20 × 100 μm space (S4A Fig) with random (*x*, *y*, *z*) coordinates. At that (initial) moment, a remaining G1-phase duration for each G1 nucleus/soma was set at 9 × (100 − *z*_*i*_) ⁄ 100 h, where *z*_*i*_ represents the distance between the center of nucleus/soma and the apical surface. Although the composition of these initial in silico VZ cells (i.e., occupied by only G1-phase and G2-phase cells, with no S- or M-phase cells) did not reflect the real value obtained in vivo [42], our calculation over about 100,000 steps achieved equilibrium with regard to the composition of cells of different cell cycle phases, as follows: 4.4 ± 1.7% for G2 phase, 2.9 ± 2.1% for M phase, 43.0 ± 7.3% for G1 phase, 11.7 ± 4.7% for S phase, and 24.2 ± 3.3% for differentiated/cell cycle exited, consistent with Takahashi et al. [3, 42]. All data analysis was performed between steps 350,000 to 400,000 of the calculation.

A pioneering version of the “virtual VZ” simulation was performed by Kosodo et al. [26] in the neocortical VZ. Based on results of comprehensive monitoring and quantification of all VZ cells’ nuclear movements in slices prepared from H2B-mCherry mice [18], we made two technical modifications for improvement: (1) we restricted the horizontal/lateral displacement of all nuclei/somata in VZ (as shown in S4A Fig) and (2) we carefully compared the apicobasal movements of nuclei between the real VZ cells and our virtual VZ using the MSD. Another modification that we made was that while Kosodo et al. [26] described the amount of soma–soma repulsions to be constant, our simulation set the repulsive force to have an inverse relationship with the distance between nuclei/somata (S4A Fig).

Because our simulation described the repulsion between nuclei/somata as a first-order approximation in Eq 6 (see also S4A Fig), the physical properties of actual cells derived from cortical surface tension [59] is partially included. However, the measurement of physical properties such as viscoelasticity, which is (1) an indispensable factor to consider the actual mode of repulsion between nucleus/somata and (2) highly related with cell-surface tension, cytoskeletal organizations, and, probably, cell shapes in tissue, is still challenging [60].

## Supporting information

S1 Fig Model: Elasticity-based passive nucleokinesis from the Windkessel-like subapical space. (A) Schematic illustrations of Windkessel mechanisms utilized by firemen for water discharge or by the aorta for forwarding blood. (B and C) Two physical models depicting the passive displacement of a plastic ball by elastic recoiling of rubber strings (B) or tubes (C). While the ball is pushed downward by a finger (cyan arrow), mechanical stress is stored in the strings or tubes, especially in the portion surrounding the lower half of the ball and very close to the bottom plate/mesh to which the strings/tubes are fixed. Upon termination of pushing (removal of the finger), the ball is propelled upward (magenta arrow) through elastic recoil of the prestressed (Windkessel-like) strings/tubes.
(TIF)

S2 Fig Assessment of stability, cytoskeletal properties, and dynamics of the apical surface and the subapical space. (A) Oblique view of a snapshot of VZ cells’ apices (green) automatically contour extracted from a time-lapse series of the en face–imaged apical surface of a cerebral wall prepared from an E13 ZO1-EGFP Tg mouse. The normal vector (red) to each apex was used to calculate the inner products (*x* axis of the graph in Fig 3D) during monitoring. If unit vectors **n1** and **n2** obtained at two adjacent time points are parallel, their inner product **n1**∙**n2** (= cos*θ*, where *θ* is the angle between **n1** and **n2**) will be 1. (B, C) Formation of a protrusion from the apical surface after somal laser ablation. See Materials and methods for details. (D) Shrinking of laser-ablated soma without losing the continuity of the plasma membrane. (E–H) Anti–α-tubulin (green) and phalloidin (magenta) staining showed that microtubules and F-actin were abundant in the VZ cells’ apical processes, which are densely distributed within the subapical space and the apical surface, including closely surrounding M-phase cells’ somata. (I) Oblique view of the subapical space (green) and its components, soma of an M-phase cell (magenta) and non–M-phase cells’ apical processes (cyan). (J) Myosin II–dependent motility of the lammelipodia-like protrusions. Scale, 5 μm in B, C, D, and J; 10 μm in E–I. E, embryonic day; VZ, ventricular zone.
(TIF)

S3 Fig In silico, pharmacological, and mechanical evaluation of the elasticity-based mechanism for daughter cells’ initial nucleokinesis. (A) Parameters for the mathematical simulation of movements of the nucleus/soma during the transition from G2 to early G1 phase in the subapical space. See Supplemental Experimental Procedures for details. (B) Graph showing the relationship between the initial spring length at stress-free state (*l*_0_) of the elastic strings and the trajectory of early G1-phase cells’ nuclei/somata. (C) In vivo immunohistochemistry showing ectopic pH3^+^ M-phase somata induced by blebbistatin. (D and E) Graphs comparing the basal nuclear/somal displacement between control (DMSO treated) and myosin II–inhibited (20 μm blebbistatin) daughter cells. Both the initial quick phase (within 30 min after cytokinesis/birth) and the next slower phase (from 30 min to 60 min) of basal nucleokinesis were affected. (F) Decompressing subapical laser ablation circularly performed around a pair of newborn daughter cells. Immediate (within 10 s) shrinkage (circularly darkened) was observed in the subapical space. Images were acquired at 5-s intervals. See Fig 6B and 6C for the effect of this decompressing ablation on the daughter cells’ nucleokinesis. (G) Compression of a cerebral wall freshly prepared from an H2B-mCherry mouse embryo using a silicon-rubber chamber (Fig 6D). Narrowing of the chamber occurred horizontally in the left panel, the entire field en face imaged subapically (at 5 μm deep). PIV in two ROIs (right panels) shows that the central portions were centripetally compressed (receiving arrows). Similar centripetal deformations were consistently reproduced when we set different ROIs, indicating that compression was achieved almost evenly/homogeneously within the entire subapical space. (H) Anti-Sox2 immunostaining of a VZ cell that was singly compressed by a capillary during “imprint” preparation, showing that the compressed cell was a progenitor cell. Scale, 10 μm in F and H; 50 μm in C and G. Underlying data can be found in S1 Data. *d*, lateral distance between the strings; *L*, apicobasal distance between the fixed ends of the elastic strings; PIV, particle image velocimetry; ROI, region of interest; VZ, ventricular zone; ***σ***, radius of the nucleus/soma.
(TIF)

S4 Fig Neuroepithelium virtually reproduced in silico and ecologically dissected, focusing mechanical relationships between M-phase and non–M-phase cells. (A) Conditions assigned for positioning nuclei/somata in the mathematical model. Nuclei/somata were defined to be repulsive to each other, with force dependent upon distance (α) (left upper panel). Cell cycle phase–dependent forces were given apically or basally to nuclei/somata in G2 phase or early G1 phase (<10 μm), respectively (right panel). *ϕ* represents time or the cell cycle phase of a given cell’s nucleus/soma. Tangential displacements (along the *x* and *y* axes) were restricted based on the horizontal bundling of apicobasally elongated neuroepithelial cells (left lower panel). See Materials and methods for details. (B and C) MSD of nuclei/somata in G1 (B, *n* = 20) and G2 (C, *n* = 10) phases in the simulation, which reproduced patterns observed in vivo [18]. (D) Snapshot of the virtual neuroepithelium, showing that the frequency of direct contacts between nuclei/somata (color coded) in the subapical space (blue) was low. (E and F) Graphs of the trajectory (E) and MSD (F) of nuclei/somata of newborn daughter cells show that the initial (within 30 min, green) phase was quicker and more directional than the subsequent (30–60 min, blue) phase (F, *n* = 6 pairs). (G) Time series visualizing the acceleration (color coded) of a daughter cell moved from birth (t = 0) until it reached a basal part of VZ, showing that high acceleration occurs only in the initial step of the successful basalward IKNM. (H) Physical give-and-take relationships between M-phase and non–M-phase cells, as revealed in this and our previous study [18]. Each M-phase cell not only gives its basal process to one daughter cell but also gives mechanical energy to both daughter cells, with elastic assistance from the densely packed apical processes of neighboring non–M-phase cells. These mother-to-daughter (intra-clonal) physical gifts assist daughter cells’ prompt nucleosomal movement away from the subapical space. Thus, such established initial basal nucleokinesis enables non–M-phase cells to have thin and flexible apical processes throughout the subapical space, which is permissive for the voluminous division of new M-phase cells. This physical cooperation may underlie efficient and safe intra-neuroepithelial nuclear/somal logistics and protect the subapical space from local overcrowding. Underlying data can be found in S1 Data. IKNM, interkinetic nuclear migration; MSD, mean-squared displacement; VZ, ventricular zone.
(TIF)

S1 Movie Time-lapse observation of IKNMs exhibited by a single NPC labeled with lynN-EGFP and H2B-RFP and its daughter cells. A singly visualized NPC (green, plasma membrane; magenta, nucleus) divided at the apical surface, giving rise to two daughter cells. IKNM, interkinetic nuclear migration; NPC, neural progenitor cell.
(MOV)

S2 Movie Departure of clonal daughter cells’ nuclei that had a direct contact with another G2/M soma. Departure of newborn daughter cell clone (cyan arrowheads) was monitored under en face horizontal sectional observation (at 5 μm from the apical surface) of an E13 cerebral wall of H2B-mCherry and Lyn-Venus double-transgenic mice. Summarized in Fig 2C. E, embryonic day.
(MOV)

S3 Movie Departure of clonal daughter cells’ nuclei that did not have direct soma–soma contacts with other G2/M somata. Departure of newborn daughter cell clone (cyan arrowheads) was monitored under en face horizontal sectional observation (at 5 μm from the apical surface) of an E13 cerebral wall of H2B-mCherry and Lyn-Venus double-transgenic mice. Summarized in Fig 2D. E, embryonic day.
(MOV)

S4 Movie Two physical models depicting the passive displacement of a plastic ball by elastic recoiling of rubber strings or tubes. While the ball is pushed downward by a finger, mechanical stress is stored in the strings (left) or tubes (right), especially in the portion surrounding the lower half of the ball and very close to the bottom plate/mesh to which the strings/tubes are fixed. Upon termination of pushing (removal of the finger), the ball is propelled upward through elastic recoil of the prestressed (Windkessel-like) strings/tubes. Summarized in S1B and S1C Fig.
(MOV)

S5 Movie Laser ablation demonstrating contractility of the apical surface. Apical surface of E13 cerebral walls that had been subjected to electroporation with ZO1-EGFP at E12 was en face inspected, and a vertex in the ZO1-EGFP–labeled mesh was severed by a short pulse of laser (yellow encircled). Images were acquired at 1-s intervals. Centrifugal movement of the surrounding vertices was tracked, and results are summarized in Fig 3B. E, embryonic day.
(MOV)

S6 Movie Time-lapse imaging of a single H2B-RFP–labeled clone in a slice prepared from an E13 ZO1-EGFP mouse. A cerebral wall prepared from an E13 ZO1-EGFP transgenic mouse was monitored (right, en face projected view; left orthogonally projected view) at 1-min intervals. En face images sequentially obtained along the apicobasal axis (over 30 μm from the surface, 0.75-μm pitch) were reconstructed. A progenitor divided to give rise to two daughter cells. The apical surface was stable at the site of cell division. Summarized in Fig 3C. E, embryonic day.
(MOV)

S7 Movie Extraction of cell borders at the apical surface and visualization of normal vectors for assessing the stability of apices. Individual cell contours (green) were extracted from a movie obtained by en face observation of the apical surface of a cerebral wall prepared from a ZO1-EGFP transgenic mouse. Normal vectors to the apices are shown red (see also S2A Fig). Stability (lack of tilting) of the normal vectors is summarized in Fig 3D.
(MOV)

S8 Movie Centripetal recoiling of the subapical surface immediately after laser ablation of a M-phase cell. Under en face horizontal sectional observation (at 5 μm from the apical surface) of an E13 cerebral wall live stained with FM4-64, a pulse of laser was applied to the center of the M-phase cell, followed immediately by shrinkage of the M-phase cell and centripetal recoiling of the surrounding cellular elements. Images were acquired at 1-s intervals. Summarized in Fig 3F. E, embryonic day.
(MOV)

S9 Movie Centripetal recoiling of the subapical surface immediately after laser ablation of a clonally labeled M-phase cell. Under en face horizontal sectional observation (at 5 μm from the apical surface) of an E13 cerebral wall, a pulse of laser was applied to the center of the M-phase cell (magenta encircled) visualized by LynN-mCherry, followed immediately by shrinkage of the M-phase cell and centripetal recoiling of the surrounding cellular elements. Images were acquired at 1-s intervals. Summarized in Fig 3G. E, embryonic day.
(MOV)

S10 Movie Subapical-specific lamellipodia-like protrusions observed in slice culture. An apical process of a slice-cultured VZ cell extending dynamically lamellipodia-like protrusions only in the subapical space (within 5 μm from the apical surface), acquired at 8-s intervals. En face images sequentially obtained along the apicobasal axis (over 18 μm from the surface, 0.75-μm pitch) were reconstructed. Summarized in Fig 3I. VZ, ventricular zone.
(MOV)

S11 Movie Myosin II–dependent motility of the lammelipodia-like protrusions. Under en face horizontal sectional observation (at 5 μm from the apical surface, acquired at 1-min intervals) of an apical process, treatment with 20 μm blebbistatin for 90 min resulted in the reduced motility of lammelipodia-like protrusions. Summarized in S2J Fig.
(MOV)

S12 Movie Three-dimensional structure of the subapical-specific lamellipodia-like protrusions, as revealed by SBF-SEM. Three apical processes in the subapical space in an E13 cerebral wall were reconstructed by serial block-face SEM. A representative cross-sectional view is shown in Fig 3J. E, embryonic day; SBF-SEM, serial block-face scanning electron microscopy.
(MOV)

S13 Movie Mathematical simulation showing a bouncing-like basal displacement of a G1-phase cell’s nucleus/soma following apical and lateral pushing of the subapical space by the mother cell. Two strings, fixed to the apical surface, behave elastically, mimicking the densely process-filled subapical space of VZ, with force storage (red in color code [during M phase]) and subsequent release (during early G1). See also Fig 4A and 4B; S3A and S3B Fig. VZ, ventricular zone.
(MOV)

S14 Movie Three-dimensional time-lapse imaging of a clonally Lifeact-EGFP–labeled progenitor cell and its daughter cells. En face images sequentially obtained along the apicobasal axis (over 20 μm from the surface, 0.5-μm pitch, 1-min intervals) were 3D reconstructed. Initially (during G2 phase), F-actin was enriched at the apical junction. During the G2-to-M transition (about 60 min), F-actin accumulated near the basal pole of the soma, followed immediately (within 80 min) by thinning of the basal process and subsequently (about 120 min) by rounding up of the soma. F-actin accumulation at the basal side continued during the growth of the cleavage furrow in a basal-to-apical direction (about 140 min) and then became undetectable after completion of cytokinesis. See also Fig 4C and 4D.
(MOV)

S15 Movie Two more examples of 3D time-lapse imaging of a single Lifeact-EGFP–labeled progenitor cell and its daughter cells. En face images sequentially obtained along the apicobasal axis (over 20 μm from the surface, 0.5-μm pitch, 1-min intervals) were 3D reconstructed. Quantitative analyses of F-actin–accumulation and the morphological changes are shown in Fig 4D.
(MOV)

S16 Movie Myosin II inhibition resulted in basal bouncing of M-phase cell’s somata. Cross-sectional imaging (5-min intervals) of cerebral wall from E13 H2B-mCherry Tg mice showing abnormal basal bouncing of M-phase cells’ somata (magenta asterisk) upon blebbistatin (20 μm) treatment. Summarized in Fig 4F. E, embryonic day.
(MOV)

S17 Movie Three-dimensionally reconstructed time-lapse observations of a representative pair of sister daughter cells labeled with LynN-EGFP and H2B-RFP. En face images sequentially obtained along the apicobasal axis (over 20 μm from the surface, 0.5-μm pitch, 2.5-min intervals) were 3D reconstructed (left). Sectional area of the cells at 5 μm from the apical surface (right) showed continuous reduction, even before the onset of basalward nucleokinesis. Summarized in Fig 5A.
(MOV)

S18 Movie Laser ablation of the process-rich subapical space inhibited the initial basal displacement of daughter cells’ nuclei/somata. Under en face horizontal sectional observation (at 5 μm from the apical surface, 5-min intervals) of an E13 cerebral wall stained with FM4-64, a pulse of laser was simultaneously applied at 15 points in a circle around the newborn daughter cells (right), which resulted in the inhibition of their initial basal displacement. The left image shows a control experiment. Results are summarized in Fig 6B and 6C.
(MOV)

S19 Movie Laser ablation-induced shrinkage of the process-rich subapical space. Under en face horizontal sectional observation (at 5 μm from the apical surface, 5-s intervals) of an E13 cerebral wall stained with FM4-64, a pulse of laser was simultaneously applied at 15 points in a circle (magenta dots) around the newborn daughter cells, followed by the immediate shrinkage of the process-rich subapical space. Summarized in S3F Fig. E, embryonic day.
(MOV)

S20 Movie Experimentally performed unidirectional centripetal compression of a cerebral wall. Under en face horizontal sectional observation (at 5 μm from the apical surface, 5-min intervals), E13 cerebral wall from H2B-mCherry Tg were compressed as shown in Fig 6D, followed by unidirectional centripetal compression with increased nuclear density. Summarized in Fig 6E. E, embryonic day.
(MOV)

S21 Movie Initial nucleokinesis of daughter cells’ nuclei in the control subapical space. En face horizontal sectional observation (at 5 μm from the apical surface, 5-min intervals) of E13 cerebral wall from H2B-mCherry Tg showing the time between the mother cell’s division (magenta arrowhead) and departure of daughter-cell nuclei (E-IKNM, cyan arrowhead; L-IKNM, yellow arrowhead). Summarized in Fig 6F. E-IKNM, early-nucleokinetic; L-IKNM, late-nucleokinetic.
(MOV)

S22 Movie Earlier initial nucleokinesis of daughter cells’ nuclei in the compressed subapical space. En face horizontal sectional observation (at 5 μm from the apical surface, 5-min intervals) of the compressed cerebral wall showing earlier initial nucleokinesis of both daughter cells’ nuclei (E-IKNM, cyan arrowhead; L-IKNM, yellow arrowhead). Results are summarized in Fig 6G and 6H. E-IKNM, early-nucleokinetic; L-IKNM, late-nucleokinetic.
(MOV)

S23 Movie Mathematically simulated IKNM and a virtual VZ. (Left) Simulation of a single clone from S phase (white) to M phase (magenta), and further to the two daughter cells’ basal nucleokinesis (initially green during G1, turning white [entered S phase] and orange [exited from cell cycle = differentiated]). See also Fig 7A and S4A Fig. (Right) Nuclei/somata of all VZ cells individually move in a cell cycle–dependent manner (shown at 6-min intervals), recapitulating a pseudostratified structure. S→ G2 → M → G1 phases are shown in white → magenta → yellow → green. Differentiated (cell cycle–exited) cells are orange. Summarized in Fig 7B. IKNM, interkinetic nuclear migration; VZ, ventricular zone.
(MOV)

S24 Movie Visualization of the degree of acceleration of a single daughter cell’s nucleus/soma in the virtual VZ simulation. Acceleration (color coded) is high (red) in the subapical space, while low (blue) in more basal regions, of the virtual VZ. Summarized in S4G Fig. VZ, ventricular zone.
(MOV)

S25 Movie Virtual VZ mathematically simulated without an initial basalward propelling force in the early G1 nuclei/somata. In silico removal (from t = 0 min) of the initial basalward propelling force resulted in unsuccessful basal displacement of early G1 daughter cells’ nuclei/somata from the subapical space (left) and disruption of the virtual neuroepithelial structure (right). Summarized in Fig 7E and 7F. VZ, ventricular zone.
(MOV)

S26 Movie Formation of a protrusion from the apical surface after somal laser ablation. En face images of a M-phase cell with laser ablation at the center of soma (magenta circle) were sequentially obtained along the apicobasal axis (over 15 μm from the surface, 2-μm pitch, 5-s intervals), followed by 3D reconstruction. A protrusion was formed from the apical surface in parallel with the shrinkage of soma. Summarized in S2B Fig. See also Materials and methods for details.
(MOV)

S1 Data Set of raw data used in this manuscript. (XLSX)

## Abbreviations

AFM: atomic force microscopy
E: embryonic day
E-IKNM: early-nucleokinetic
IKNM: interkinetic nuclear migration
L-IKNM: late-nucleokinetic
MSD: mean-squared displacement
NE: neuroepithelium
NPC: neural progenitor cell
PLP: periodate-lysine-paraformaldehyde
SBF-SEM: serial block-face scanning electron microscopy
VZ: ventricular zone
