# Supplementary figures and images for: Elasticity-based boosting of neuroepithelial nucleokinesis via indirect energy transfer from mother to daughter
Source: PLoS Biol. 2018 Apr 20;16(4):e2004426. doi: 10.1371/journal.pbio.2004426 (PMC5931692; doi:10.1371/journal.pbio.2004426)

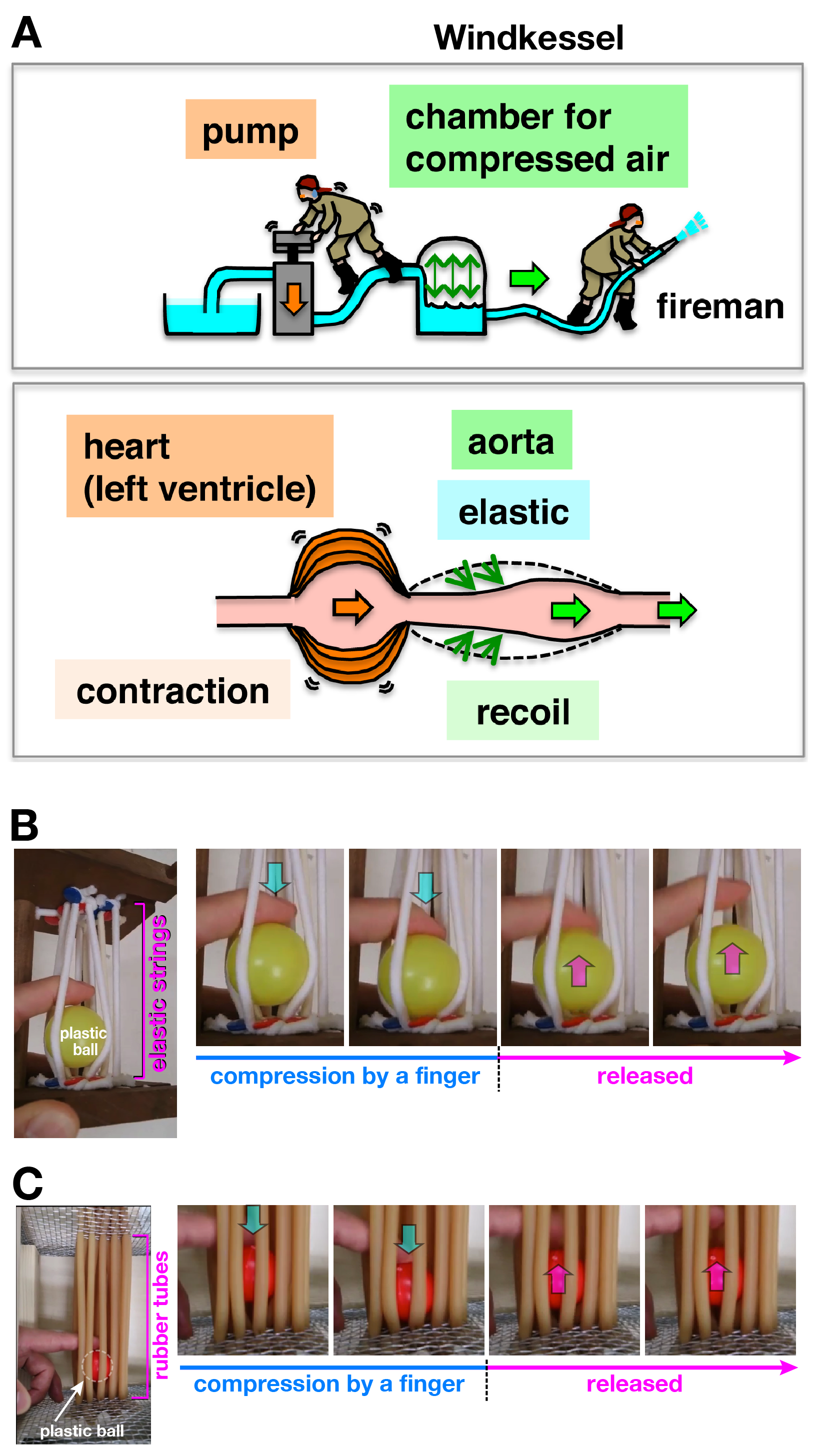

Supplement: S1 Fig — (A) Schematic illustrations of Windkessel mechanisms utilized by firemen for water discharge or by the aorta for forwarding blood. (B and C) Two physical models depicting the passive displacement of a plastic ball by elastic recoiling of rubber strings (B) or tubes (C). While the ball is pushed downward by a finger (cyan arrow), mechanical stress is stored in the strings or tubes, especially in the portion surrounding the lower half of the ball and very close to the bottom plate/mesh to which the strings/tubes are fixed. Upon termination of pushing (removal of the finger), the ball is propelled upward (magenta arrow) through elastic recoil of the prestressed (Windkessel-like) strings/tubes. (TIF) [file pbio.2004426.s001.tif]

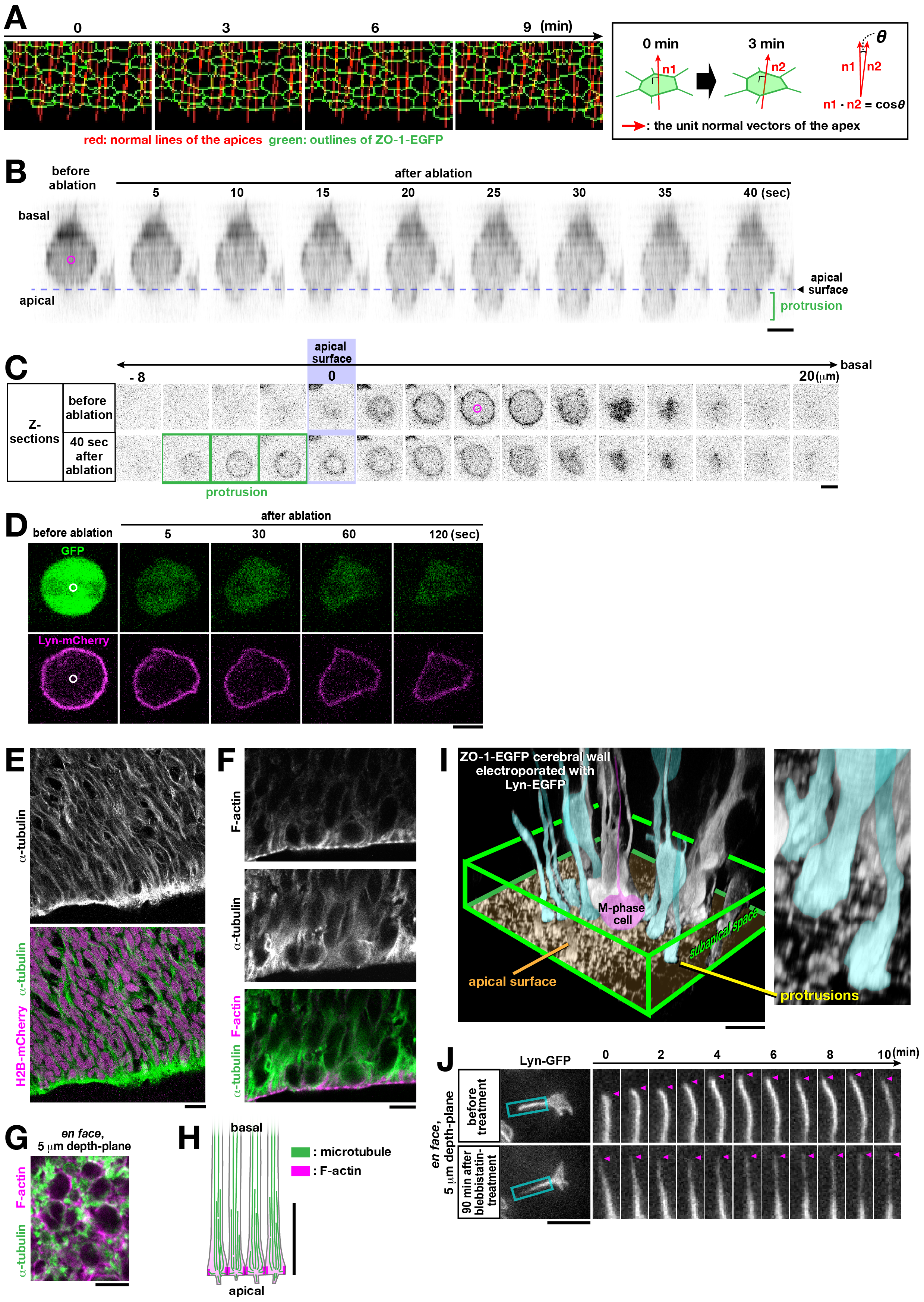

Supplement: S2 Fig — (A) Oblique view of a snapshot of VZ cells’ apices (green) automatically contour extracted from a time-lapse series of the en face–imaged apical surface of a cerebral wall prepared from an E13 ZO1-EGFP Tg mouse. The normal vector (red) to each apex was used to calculate the inner products (x axis of the graph in Fig 3D) during monitoring. If unit vectors n1 and n2 obtained at two adjacent time points are parallel, their inner product n1∙n2 (= cosθ, where θ is the angle between n1 and n2) will be 1. (B, C) Formation of a protrusion from the apical surface after somal laser ablation. See Materials and methods for details. (D) Shrinking of laser-ablated soma without losing the continuity of the plasma membrane. (E–H) Anti–α-tubulin (green) and phalloidin (magenta) staining showed that microtubules and F-actin were abundant in the VZ cells’ apical processes, which are densely distributed within the subapical space and the apical surface, including closely surrounding M-phase cells’ somata. (I) Oblique view of the subapical space (green) and its components, soma of an M-phase cell (magenta) and non–M-phase cells’ apical processes (cyan). (J) Myosin II–dependent motility of the lammelipodia-like protrusions. Scale, 5 μm in B, C, D, and J; 10 μm in E–I. E, embryonic day; VZ, ventricular zone. (TIF) [file pbio.2004426.s002.tif]

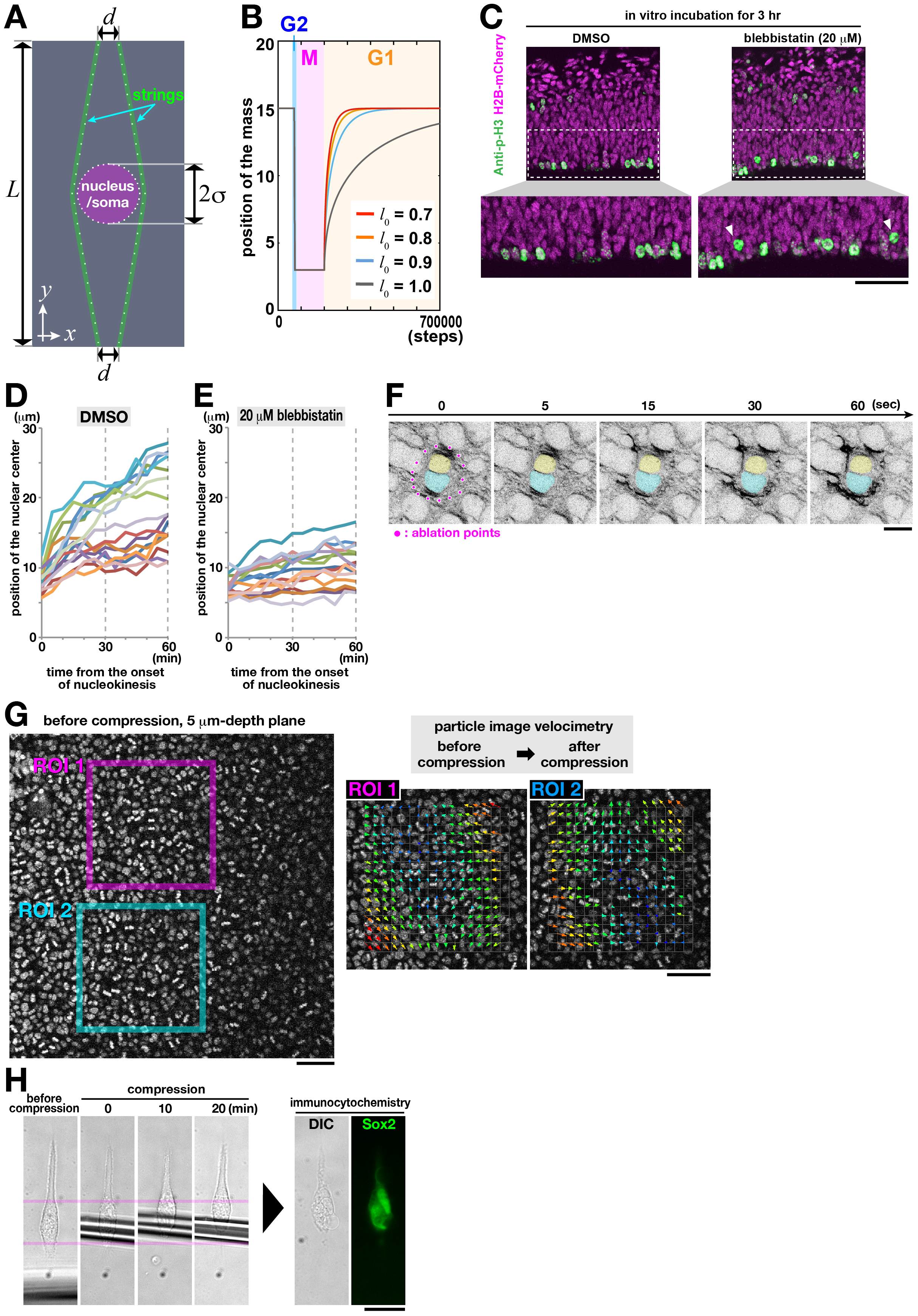

Supplement: S3 Fig — (A) Parameters for the mathematical simulation of movements of the nucleus/soma during the transition from G2 to early G1 phase in the subapical space. See Supplemental Experimental Procedures for details. (B) Graph showing the relationship between the initial spring length at stress-free state (l0) of the elastic strings and the trajectory of early G1-phase cells’ nuclei/somata. (C) In vivo immunohistochemistry showing ectopic pH3+ M-phase somata induced by blebbistatin. (D and E) Graphs comparing the basal nuclear/somal displacement between control (DMSO treated) and myosin II–inhibited (20 μm blebbistatin) daughter cells. Both the initial quick phase (within 30 min after cytokinesis/birth) and the next slower phase (from 30 min to 60 min) of basal nucleokinesis were affected. (F) Decompressing subapical laser ablation circularly performed around a pair of newborn daughter cells. Immediate (within 10 s) shrinkage (circularly darkened) was observed in the subapical space. Images were acquired at 5-s intervals. See Fig 6B and 6C for the effect of this decompressing ablation on the daughter cells’ nucleokinesis. (G) Compression of a cerebral wall freshly prepared from an H2B-mCherry mouse embryo using a silicon-rubber chamber (Fig 6D). Narrowing of the chamber occurred horizontally in the left panel, the entire field en face imaged subapically (at 5 μm deep). PIV in two ROIs (right panels) shows that the central portions were centripetally compressed (receiving arrows). Similar centripetal deformations were consistently reproduced when we set different ROIs, indicating that compression was achieved almost evenly/homogeneously within the entire subapical space. (H) Anti-Sox2 immunostaining of a VZ cell that was singly compressed by a capillary during “imprint” preparation, showing that the compressed cell was a progenitor cell. Scale, 10 μm in F and H; 50 μm in C and G. Underlying data can be found in S1 Data. d, lateral distance between the strings; L, apicobasal dis [file pbio.2004426.s003.tif]

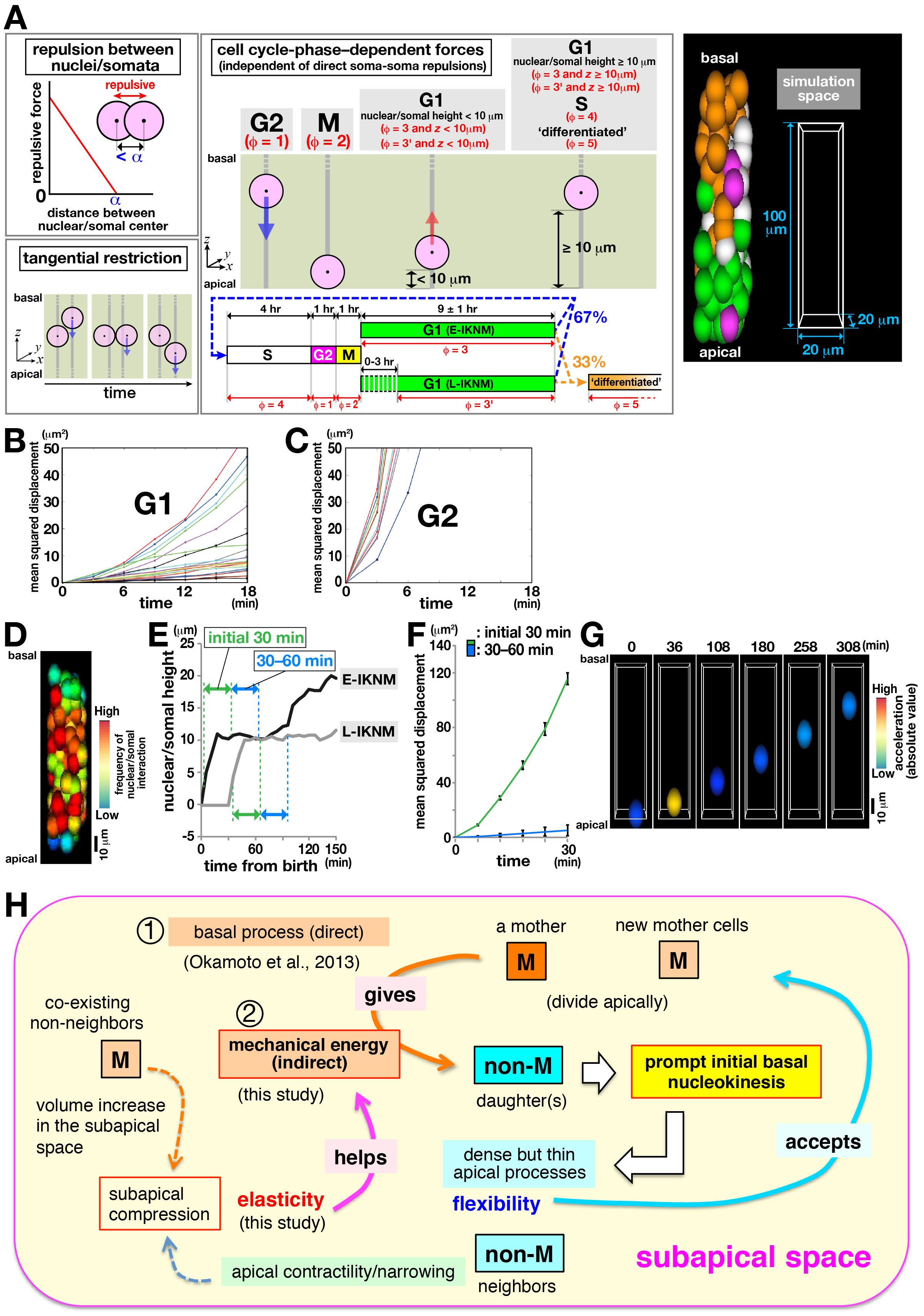

Supplement: S4 Fig — (A) Conditions assigned for positioning nuclei/somata in the mathematical model. Nuclei/somata were defined to be repulsive to each other, with force dependent upon distance (α) (left upper panel). Cell cycle phase–dependent forces were given apically or basally to nuclei/somata in G2 phase or early G1 phase (<10 μm), respectively (right panel). ϕ represents time or the cell cycle phase of a given cell’s nucleus/soma. Tangential displacements (along the x and y axes) were restricted based on the horizontal bundling of apicobasally elongated neuroepithelial cells (left lower panel). See Materials and methods for details. (B and C) MSD of nuclei/somata in G1 (B, n = 20) and G2 (C, n = 10) phases in the simulation, which reproduced patterns observed in vivo [18]. (D) Snapshot of the virtual neuroepithelium, showing that the frequency of direct contacts between nuclei/somata (color coded) in the subapical space (blue) was low. (E and F) Graphs of the trajectory (E) and MSD (F) of nuclei/somata of newborn daughter cells show that the initial (within 30 min, green) phase was quicker and more directional than the subsequent (30–60 min, blue) phase (F, n = 6 pairs). (G) Time series visualizing the acceleration (color coded) of a daughter cell moved from birth (t = 0) until it reached a basal part of VZ, showing that high acceleration occurs only in the initial step of the successful basalward IKNM. (H) Physical give-and-take relationships between M-phase and non–M-phase cells, as revealed in this and our previous study [18]. Each M-phase cell not only gives its basal process to one daughter cell but also gives mechanical energy to both daughter cells, with elastic assistance from the densely packed apical processes of neighboring non–M-phase cells. These mother-to-daughter (intra-clonal) physical gifts assist daughter cells’ prompt nucleosomal movement away from the subapical space. Thus, such established initial basal nucleokinesis enables non–M-phase cells to have thin an [file pbio.2004426.s004.tif]
